# Prevalent, persistent anal HPV infection and squamous intraepithelial lesions: Findings from a cohort of men living with HIV in South Africa

**DOI:** 10.1371/journal.pone.0225571

**Published:** 2019-12-05

**Authors:** Admire Chikandiwa, Pedro. T. Pisa, Catherine Tamalet, Etienne. E. Muller, Pamela Michelow, Matthew. F. Chersich, Philippe Mayaud, Sinead Delany-Moretlwe

**Affiliations:** 1 Wits RHI, Faculty of Health Sciences, University of the Witwatersrand, Johannesburg, South Africa; 2 Department of Clinical Microbiology IHU and CNRS-URMITE, UMR 7278 Timone University Hospital Marseille, France; 3 National Institute for Communicable Diseases, National Health Laboratory Service, Johannesburg, South Africa; 4 Cytology Unit, Department of Anatomical Pathology, University of the Witwatersrand and National Health Laboratory Services, Johannesburg, South Africa; 5 London School of Hygiene and Tropical Medicine, London, United Kingdom; Katholieke Universiteit Leuven Rega Institute for Medical Research, BELGIUM

## Abstract

**Objective:**

To estimate the prevalence, incidence and persistence of anal HPV infection and squamous intra-epithelial lesions (SILs) among men living with HIV (MLHIV), and determine their risk factors.

**Methods:**

We enrolled MLHIV ≥18 years, who attended 6-monthly visits for 18 months. Socio-behavioural data were collected by questionnaire. Clinicians collected blood sample (CD4+ count and HIV plasma viral load), anal swabs (HPV DNA testing) and anal smears (Bethesda classification) at each visit. HPV DNA testing and classification of smears were done at enrolment and last follow-up visit (two time points). Factors associated with persistent anal HPV infection and SILs were evaluated with generalized estimating equations logistic regression and standard logistic regression respectively.

**Results:**

Mean age of 304 participants was 38 (Standard Deviation, 8) years; 25% reported >1 sexual partner in the past 3 months. Only 5% reported ever having sex with other men. Most (65%) participants were taking antiretroviral treatment (ART), with a median CD4+ count of 445 cells/μL (IQR, 328–567). Prevalence of any-HPV infection at enrolment was 39% (88/227). In total, 226 men had anal HPV DNA results at both enrolment and final visits. Persistence of any-anal HPV infection among 80 men who had infection at enrolment was 26% (21/80). Any persistent anal HPV infection was more frequent among MLHIV with low CD4+ count (<200 vs. >500 cells/μL; aOR = 6.58; 95%CI: 2.41–17.94). Prevalence of anal SILs at enrolment was 49% (118/242) while incidence of SILs among MLHIV who had no anal dysplasia at enrolment was 27% (34/124). Of the 118 men who had anal dysplasia at enrolment, 15% had regressed and 38% persisted by month 18. Persistent anal HPV infection was associated with persistent SILs (aOR = 2.95; 95%CI: 1.08–10.89). ART status or duration at enrolment were not associated with persistent anal HPV infection or persistent SILs during follow-up.

**Conclusion:**

In spite of a high prevalence of anal HPV, HIV-positive heterosexual men have a low burden of anal HPV related disease. HPV vaccine and effective ART with immunological reconstitution could reduce this burden of infection.

## Introduction

Anogenital human papillomavirus (HPV) is the most common sexually transmitted infection worldwide and responsible for a large burden of disease.[1] High-risk (HR) HPV genotypes cause approximately 95% of the estimated 35 000 incident anal cancer cases that occur globally each year.[2] Persistence of these high-risk infections is a critical step in the progression to invasive cancer.[3]

Men living with HIV (MLHIV) have a high prevalence of HPV infection and are more likely to be infected with multiple high-risk types compared to HIV-negative men.[4] HIV co-infection increases the prevalence, persistence and progression to pre-neoplastic squamous intraepithelial lesions (SILs).[5, 6] This is possibly due to immunosuppression which impairs the clearance of HPV infections.[7]

The natural history of cervical cancer and progression from HPV infection to development of invasive cervical disease has been well documented.[8] The slow progression, which can take a median of 30 years from infection with HR-HPV types to peak incidence of invasive cancer, makes cervical cancer amenable to prevention by screening strategies.[9] By comparison, relatively little is known about the natural history of anal HPV infection and anal dysplasia in men, especially among MLHIV [10]. The role of screening to prevent anal cancer is also unclear, as evidence suggests that rates of progression of SILs are lower, and more SILs regress spontaneously than is observed with cervical SIL.[11] Whilst HPV viral load (VL) has been shown to be predictive of high grade cervical lesions, its role in predicting persistent and progression of anal dysplasia has not been extensively evaluated. [12] The impact of antiretroviral therapy (ART) on progression of HPV infection to disease is not clear. Some, but not all, studies report that ART reduces incidence of SILs. This could be possibly explained by differences such as the nadir CD4+ count and timing of ART initiation. [13, 14] The timing of ART initiation is particularly important in low and middle income countries (LMICs), where MLHIV usually present later for ART initiation than women [15], and men are less likely to be on treatment and have poor retention rates compared to women.[16]

To help inform the selection of future HPV prevention interventions among MLHIV in South Africa, we established a cohort study of HIV-positive men. We estimated the prevalence, persistence and incidence of anal HPV infections and SILs. We further evaluated the effect of factors such as ART status, duration on ART, CD4+ cell count and HIV plasma viral load (PVL) at baseline on these outcomes.

## Methods

### Study population and procedures

Full details of the cohort study and prevalence of HPV and SIL at enrolment have been published elsewhere.[17] Briefly, 304 HIV seropositive men aged 18 years or older who reported sexual activity in the 3 months prior to enrolment were recruited from ART treatment clinics inner-city Johannesburg.[17] Participants were enrolled and followed-up every 6 months for up to 18 months. Data on socio-demographic, behavioural and clinical history were collected by interviewer-administered questionnaire at each visit. Participants responded to sensitive questions on sexual behaviour using computer assisted self-interview (CASI).

Anogenital examination, including digital anorectal examination, was performed by a trained clinician at each visit. Two intra-anal specimens were collected by blindly (i.e. without anoscopy) inserting Dacron swabs three centimetres into the anal canal and removing them whilst rotating and applying pressure on the walls of the canal. One swab was used for HPV DNA testing, whilst the other was used to prepare a conventional anal smear for cytological analysis.[17] Swabs were stored at -70°C before HPV DNA testing.

Venous blood was taken at each visit to test for CD4+ cell count (FACScount, BD^™^ BD Biosciences, United States) and HIV-1 plasma viral load (PVL [Roche Taqman^®^, Roche Diagnostics, Mannheim, Germany]). The standard of care at the time was men could only start ART at CD4+ count of 350 cells/μL or less. [18]

### Laboratory methods

HPV testing was conducted on intra-anal swabs using an identical method at enrolment and at the final (i.e. last follow-up which was either month 12 or 18) visit. Testing was only done at two time points due to funding limitations. The MagNA Pure LC DNA Isolation Kit I (Roche Diagnostics, Mannheim, Germany) was used to extract HPV DNA from the swabs. HPV genotyping was determined by the Roche Linear Array assay (RLA, Roche Diagnostics, Mannheim, Germany). HPV 16 and HPV 18 VL in copies per million human cells were quantified at enrolment on samples that were positive for these types using a quantitative duplex real-time PCR method. [19] This method only allows the HPV 16, 18, and albumin gene copy number to be quantified in the same assay. The human β-globin gene served as internal control for cellular adequacy, extraction efficiency and amplification. Results for any samples with inadequate internal control were reported as invalid and excluded from further testing.

Conventional smears were performed as liquid based cytology was not available at the time that the study was undertaken. Smears were graded as either unsatisfactory for analysis, negative for intraepithelial lesion or malignancy (NILM), atypical squamous cells of undetermined significance (ASCUS), atypical squamous cells-high grade lesions cannot be ruled out (ASC-H), and low- (LSIL) or high-grade squamous intraepithelial lesions (HSIL) in accordance with the Bethesda System.[20] Smears were read independently by a cytotechnologist and one cytopathologist at the National Health Laboratory Service (NHLS) laboratory in Johannesburg, South Africa. If there was a discrepancy between these two readings, the smear was given to a second cytopathologist.

### Definition of anal HPV infection and cytological outcomes

HPV DNA infection outcomes at final visit were defined as (i) *persistence*, i.e. detection of the same HPV DNA type that was detected at enrolment, and (ii) *clearance*, i.e. absence of an HPV DNA type that was detected at enrolment, and (iii) *incident infection*, i.e. detection of any HPV DNA type that was not detected at enrolment. Groups of HPV genotypes were categorised as follows: (i) *any HPV infection*, i.e. detection of at least one of the 37 HPV genotypes that can be identified by the Roche LA; (ii) *any HR-HPV infection*, i.e. detection of at least one of the following HPV genotypes: 16, 18, 31, 33, 35, 39, 45, 51, 52, 56, 58, 59 or 68; (iii) *any LR-HPV infection*, i.e. detection of at least one of the following HPV genotypes: 6, 11, 26, 40, 42, 53, 54, 55, 61, 62, 66, 69, 70, 71, 72, 73, 81, 83, 84, IS39 or CP6108; (iv) *any alpha 7 infection*, i.e. detection of at least one of the following HR-HPV genotypes: 18, 45; 39 or 59; and *any alpha 9 infection*, i.e. detection of at least one of the following HR-HPV genotypes: 16, 31, 33, 35, 52 or 58. The HPV 16 or 18 viral loads were log transformed to base 10 to normalise distributions, as done previously.[19, 21]

*Abnormal anal cytology* was defined as a diagnosis of ASCUS or higher. Anal cytology at final visit was defined as (i) *regression* to a lower grade from a higher grade OR to normal cytology from ASCUS/ASC-H at enrolment, (ii) *persistent*, i.e. abnormal anal cytology at both enrolment and final visits or transition from ASCUS/ASC-H to LSIL, and (iii) *incident*, i.e. any abnormality not detected at enrolment. High stable CD4+ count was defined as a CD4+ count >500 cells/μL at all follow-up visits. Sustained HIV virological control was defined HIV-1 PVL <40 copies/mL at all follow-up visits. Controlled HIV disease was defined as being on ART for at least 6 months, with CD4+ count of 350 cells/μL or higher and undetectable HIV-1 PVL (i.e. <40 copies/ml).

### Statistical analysis

Descriptive statistics were used to summarize the prevalence of the anal HPV infection and SILs. Chi-square tests were run to explore differences between proportions. Persistent anal HPV infection and anal SILs were categorised as a binary outcome and reported as proportions since data were available at enrolment and final visits only. The incidence rate and 95% confidence interval (95% CI) of anal HPV infection was estimated by the Kaplan-Meier method. Person time was calculated as the time from the date of sample collection at enrolment to the date of sample collection at the final follow-up visit. Multivariate models were constructed to identify socio-demographic, sexual behaviour and HIV-related factors associated with these outcomes.

Associations between persistent anal HPV infection and exposure variables were evaluated with generalized estimating equations (GEE) logistic regression with robust standard errors (vce) to account for multiple HPV genotypes and multiple infection states (persistence and clearance) that could occur within each participant.[22] The exchangeable correlation option was used to account for within-participant correlation of the different HPV genotypes. The unit of analysis was infection, not participant, as the outcome measured was persistence of an HPV infection at follow-up visit (and participants could have more than 1 infection). Eight separate GEE models were run for each HIV-related factor adjusting for potential confounders (viz. marital status, smoking, alcohol use, age at sexual debut, number of sexual partners) associated with persistent infection in bivariate analysis at P<0.10.[23] Risk factor analysis was not conducted for incident infection due to low incidence (i.e. small numbers).

Associations between anal cytological abnormalities and exposure variables were assessed using logistic regression. Adjusted standard logistic regression models were constructed to explore independent associations between these cytological abnormalities and risk factors using the same model-building approach as described above. Analysis was performed using Stata version 13 (Stata Statistical Software, College Station. TX: Stata Corporation).

### Ethics statement

The study was approved by the Wits Human Research Ethics Committee (Reference numbers: M111191 and M160859). Written, informed consent was obtained from all study participants after full explanation of the study objectives and testing procedures.

## Results

### Study population

A full description of the study population has been published elsewhere.[17] Briefly, a total 336 MLHIV were screened and 304 (90%) men with a mean age of 38 years (SD, 8 years) were enrolled into the cohort study between March 2011 and October 2012. At enrolment, 25% of participants reported more than one sexual partner in the past three months; only 5% (n = 15) reported ever having sex with other men. The majority of the participants (65%) were already taking ART (n = 197), for a median duration of 33 months (IQR, 15–58). About half (n = 106, 54%) of those on ART were virologically suppressed and had median CD4+ count of 445 cells/μL (IQR, 328–567). Of all men, 18% were classified as having well-controlled HIV (n = 55). One participant with a palpable abnormality was referred to a colorectal surgeon for further assessment and management. The final diagnosis was anal warts. The visit completion rates at months 6, 12 and 18 were 92% (n = 279), 85% (n = 257) and 80% (n = 244), respectively. Overall, 287 (95%) of the men attended at least one follow up visit.

### Prevalent anal HPV infection and dysplasia at enrolment

The data on prevalence of anal HPV infection and cytology has been published elsewhere [17]. In brief, the prevalence of any anal HPV infection at enrolment was 39% (88/227), and the prevalence of any anal cytological abnormality was 49% (118/242) distributed as 20% (n = 48) ASCUS and29% (n = 70) LSIL but no HSIL was found. The prevalence of any HR-HPV infection was 25%. Younger age, low CD4+ count and anal dysplasia were significantly associated with any HR-HPV. The proportion of men with at least one HR type contained in the quadrivalent and nonavalent vaccines were 11% and 22% respectively. Among MSM, almost all men (93% [14/15]) had any HPV infection and 73% had any HR-HPV.

### Persistent anal HPV infection and associated factors

A total of 226 men (91% of enrolled men who accepted anal smears) had valid anal HPV DNA results at both enrolment and final visits. The median (IQR) follow-up time was 1.49 (1.34–1.50) years. Although 74% (59/80) of the men who had anal HPV infections at enrolment cleared them during follow-up, a substantial proportion, 26% (21/80) had persistent infection. The proportion of MLHIV with persistent infection when this limited to heterosexual men was 23% (15/66, data not shown in table). Levels of persistence were 17% with alpha-7 types, 13% with alpha-9 types and 17% with HPV 16 (Table 1). There was a strong inverse relationship between persistent anal HPV infection and enrolment CD4+ count. Persistence was higher among MLHIV with low CD4+ counts (i.e. <200 vs. >500 cells/μL; adjusted odds ratio [aOR] = 6.58; 95%CI: 2.41–17.94, p<0.001). Persistence was non-significantly lower among men with well controlled HIV disease at enrolment (aOR = 0.09; 95%CI: 0.01–1.20, p = 0.07) compared to those with poorly controlled disease. ART status, duration on ART and HPV VL at enrolment were not independently associated with persistent anal HPV infection (Table 2). The proportion of new (incident) infections among the 180 men who had no infection at baseline was 1% (2/180). These 180 men were followed up for a total time 253.63 person years with 2 events which gives a low incidence rate of 0.79 (0.20–3.15) per 100 person years.

**Table 1:** Persistence of intra-anal HPV infection at final visit among 226 men who have results at both enrolment and final visit.

	Positive at enrolment (N = 226)	Persistence^a^	Clearance
	n (%)	n (%)	n (%)
**Any-HPV**	80 (35)	21 (26)	59 (74)
**HR-HPV**			
Any HR types^b^	52 (23)	13 (25)	39 (75)
Alpha 7^c^	36 (15)	6 (17)	20 (83)
Alpha 9^d^	32 (14)	4 (13)	28 (87)
HPV 16	18 (8)	3 (17)	15 (83)
HPV 18	9 (4)	1 (11)	8 (89)
HPV 31	4 (2)	1 (25)	3 (75)
HPV 33	4 (2)	0 (0)	4 (100)
HPV 35	4 (2)	0 (0)	4 (100)
HPV 39	2 (1)	1 (50)	1 (50)
HPV 45	15 (6)	3 (20)	12 (80)
HPV 51	8 (4)	3 (38)	5 (62)
HPV 52	5 (2)	0 (0)	5 (100)
HPV 56	5 (2)	0 (0)	5 (100)
HPV 58	10 (4)	0 (0)	10 (100)
HPV 59	12 (5)	2 (17)	10 (83)
HPV 68	8 (4)	4 (50)	4 (50)
**LR-HPV**			
Any LR types^e^	64 (28)	13 (20)	51 (80)
HPV 6	20 (9)	8 (40)	12 (60)
HPV 11	4 (2)	1 (25)	3 (75)

^a^Detection of the same HPV DNA type that was detected at enrolment;

^b^ HR-HPV include: HPV16, 18, 31, 33, 35, 39, 45, 51, 52, 56, 58, 59, 68;

^c^Alpha-7 include: HPV 18, 39, 45 and 59;

^d^Alpha-9 include: HPV 16, 31, 33, 35, 52 and 58;

^e^LR-HPV include: HPV6, 11, 40, 42, 54, 55, 61, 62, 66, 67, 69. 70, 71, 72, 73, 81, 83, 84, IS39 and CP6108

**Table 2:** Factors associated with persistent anal HPV infection at final visit, using infections as unit of measure*.

	N = 43	Crude	P-value	Adjusted^a^	P-value
	n (Column %) or Median (IQR)	OR (95% CI)		aOR (95% CI)	
**ART status at enrolment**					
No ART	5 (11.6)	1			
ART	38 (88.4)	2.81 (0.86–9.21)	0.09	2.44 (0.74–8.09)	0.14
**Duration on ART at enrolment, months**^#^					
> 12	26 (70.3)	1		1	
6–12	8 (21.6)	0.82 (0.39–1.72)	0.61	1.12 (0.41–3.04)	0.82
< 6	3 (8.1)	0.81 (0.26–2.52)	0.72	2.05 (0.57–7.42)	0.27
**Enrolment CD4+ count (cells/μL)**					
>500	12 (27.9)	1		1	
351–500	6 (14.0)	0.65 (0.25–1.71)	0.38	1.08 (0.35–3.28)	0.90
200–350	10 (23.3)	1.33 (0.62–2.85)	0.46	**3.04 (1.42–6.49)**	**0.004**
<200	15 (35.0)	**3.22 (1.78–5.82)**	**<0.001**	**6.58 (2.41–17.94)**	**<0.001**
**High stable CD4+ count**^b^	1 (2.3)	0.09 (0.01–1.50)	0.09	0.08 (0.01–1.69)	0.10
**Undetectable HIV-1 PVL (<40 copies/mL) at enrolment**	15 (34.9)	0.66 (0.35–1.26)	0.21	0.62 (0.28–1.37)	0.27
**Sustained HIV virological control**^c^	31 (72.1)	0.49 (0.23–1.09)	0.08	0.73 (0.30–1.75)	0.47
**HIV disease control status at enrolment**^d^					
On ART, but poorly controlled	36 (83.7)	1		1	
ART naive	5 (11.6)	**0.26 (0.08–0.83)**	**0.02**	**0.30 (0.09–0.96)**	**0.04**
Well controlled	2 (4.7)	0.13 (0.01–1.20)	0.07	0.09 (0.01–1.20)	0.07

*N = 43, the total number of infections (21 men had > = 1 infection),

^#^n = 37 as six infections were in men not at ART at enrolment,

^a^Adjusted Odds Ratio (aOR). Eight separate Generalised Estimating Equations models for each HIV-related factor were adjusted for marital status, smoking, alcohol use, age at sexual debut, number of sexual partners;

^b^: CD4+ count >500 cells/μL at all follow-up visits;

^c^: HIV-1 plasma viral load <40 copies/mL at all follow-up visits;

^d^: Well controlled disease defined as on ART for >6 months, CD4+ >350 cells/μL and undetectable HIV-1 plasma viral load.

### Overall progression of anal dysplasia

**Fig 1:**
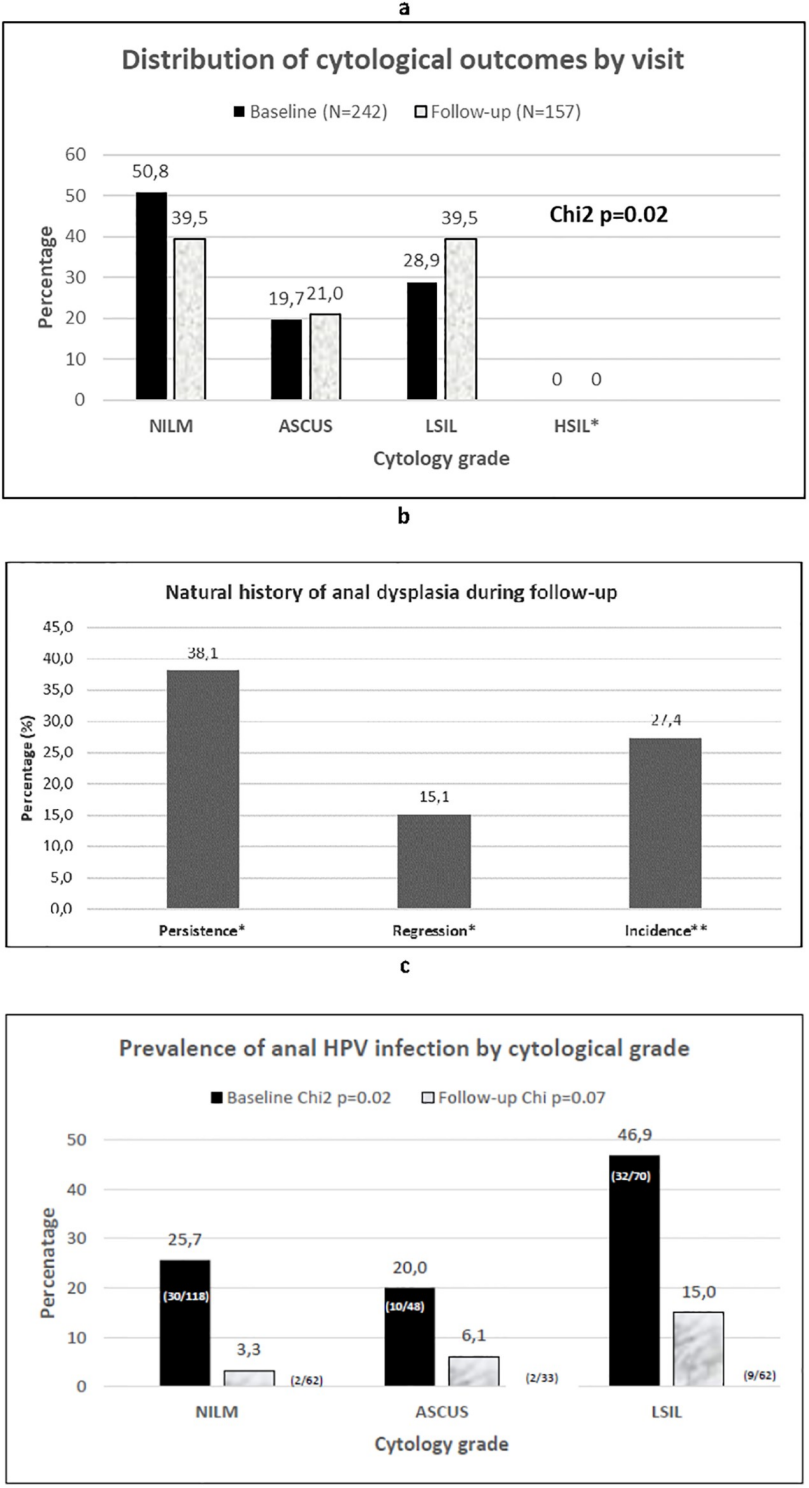
Progression of anal dysplasia. (A) Distribution of cytological outcomes by visit *(NILM*: *negative for intraepithelial lesion or malignancy; ASCUS*: *atypical squamous cells of undetermined significance; L- or H-SIL*: *low- or high-grade squamous intraepithelial lesions)*. (B) Natural history of anal dysplasia during follow-up. (C) Prevalence of anal HPV infection by cytological grade *(NILM*: *negative for intraepithelial lesion or malignancy; ASCUS*: *atypical squamous cells of undetermined significance; L- or H-SIL*: *low- or high-grade squamous intraepithelial lesions)*; *Among 118 men who had abnormal cytology at enrolment, ** Among 124 men who had normal cytology at enrolment.

Among 250 anal smears that were collected at enrolment, 8 (3.2%) were of poor quality and could not be analysed. Of the remaining 242 smears, 124 (50.8%) were graded as NILM, 48 (19.7%) were ASCUS and 70 (28.99%) were LSIL. Of the 169 smears collected at the final visit, 12 (7.1%) were excluded due to poor quality. Among the remaining 157 smears, 62 (39.5%) were graded NILM, 33 (21.0%) were ASCUS and 62 (39.5%) were LSIL. There were no cases of HSIL diagnosed at either enrolment or final visits. The proportion of smears with abnormal anal cytology was significantly higher at month 18 compared to the enrolment visit (61% vs 49%, p = 0.02) (Fig 1A).

Regression of anal dysplasia at final follow-up visit among the 118 men who had abnormal cytology at enrolment was 15% (n = 18). Most of the cases changed from LSIL to NILM (n = 10), whilst eight regressed from ASCUS to NILM. Thirty eight percent (n = 45) had persistent anal dysplasia at final follow-up visit with the majority being LSIL cases (n = 28), whilst eight cases were ASCUS and 9 cases transited from ASCUS to LSIL (Fig 1B). At final visit, 34 (27%) new cases of anal dysplasia were detected among the 124 MLHIV who had NILM at enrolment. The majority of new cases were LSIL (n = 22) with the remainder being ASCUS (n = 12). The prevalence of HPV infection varied by cytological grade and was highest for LSIL at both enrolment and month 18 (Fig 1C).

### Factors associated with persistent anal cytological abnormalities

There was some evidence of inverse relationship between persistent anal cytological abnormalities and enrolment CD4+ cell count. MLHIV who had a shorter duration of ART were more likely to have persistent anal cytological abnormalities. This was significant among men who had been on ART for 12 months or less (aOR = 2.25; 95%CI: 1.74–6.76, p = 0.05) when compared those who had been on ART for more than 12 months at enrolment.

At enrolment, HR-HPV (aOR = 3.14; 95%CI: 1.33–7.43, p = 0.009) or any alpha-9 type infections (aOR = 2.70; 95%CI: 1.25–18.09, p = 0.05) were strongly associated with persistent anal cytological abnormalities. Similarly, any persistent anal HPV (aOR = 2.95; 95%CI: 1.08–10.89, p = 0.05) and any persistent HR-HPV (aOR = 4.64; 95%CI: 1.80–14.95, p = 0.04) infections were associated with persistent anal cytological abnormalities.

ART status, HIV-1 PVL and HPV VL at enrolment were not independently associated with persistent anal cytological abnormalities (Table 3).

**Table 3:** Factors associated with persistent anal cytological abnormalities*.

Description	N = 45	Crude	P-value	Adjusted^a^	P-value
	n (column %) or Median (IQR)	OR (95% CI)		aOR (95% CI)	
**ART status at enrolment**					
No ART	13 (28.9)	1		1	
ART	32 (71.1)	1.40 (0.56–2.75)	0.37	1.27 (0.56–2.85)	0.56
**Duration on ART at enrolment (months)**^#^					
> 12	23 (24.6)	1		1	
<12	8 (42.1)	3.17 (1.17–8.61)	0.02	2.25 (1.74–6.76)	**0.05**
**Enrolment CD4+ count (cells/μL)**					
>500	14 (33.3)	1		1	
351–500	15 (35.7)	1.20 (0.52–2.79)	0.70	1.12 (0.47–2.69)	0.79
201–350	9 (21.4)	1.34 (0.50–3.58)	0.60	1.38 (0.51–3.72)	0.53
<200	4 (9.5)	1.64 (0.43–6.29)	0.50	1.62 (0.42–6.27)	0.49
**High stable CD4+ count**^b^	8 (17.8)	0.73 (0.29–1.83)	0.50	0.77 (0.30–1.96)	0.58
**Undetectable HIV-1 PVL (<40 copies/mL)**	19 (44.1)	0.84 (0.41–1.71)	0.64	0.89 (0.43–1.89)	0.77
**Sustained HIV virological control**^c^	31 (21.2)	0.97 (0.33–2.82)	0.96	0.96 (0.33–2.89)	0.96
**Disease control status at enrolment**^d^					
On ART, but poorly controlled	13 (29.6)	1		1	
ART naive	17 (38.6)	1.25 (0.55–2.87)	0.60	1.14 (0.47–2.78)	0.77
Well controlled	14 (31.8)	1.62 (0.67–3.91)	0.30	1.41 (0.55–3.65)	0.47
**Prevalent HPV infection at enrolment**					
Any-HPV	17 (38.6)	1.64 (0.80–3.40)	0.20	1.34 (0.62–2.92)	0.16
Any HR-HPV	16 (36.4)	**3.43 (1.54–7.63)**	**0.003**	**3.14 (1.33–7.43)**	**0.009**
Any alpha-7^e^	8 (18.2)	2.42 (0.89–6.60)	0,08	1.94 (0.65–5.79)	0.24
Any alpha-9^f^	9 (20.5)	**4.58 (1.16–8.54)**	**0,03**	**2.70 (1.25–18.09)**	**0.05**
HPV 16	5 (11.4)	2.92 (0.80–10.64)	0.10	2.33 (0.59–9.23)	0.23
HPV 18	4 (9.1)	2.88 (0.69–12.04)	0.15	2.16 (0.41–11.32)	0.36
HPV 45	5 (11.4)	2.92 (0.80–10.64)	0.10	2.10 (0.53–8.13)	0.29
**Persistent HPV infection**^h^					
Any-HPV	7 (15.9)	**3.56 (1.13–11.27)**	**0.03**	**2.95 (1.08–10.89)**	**0.05**
Any HR-HPV	5 (11.4)	**4.96 (1.13–21.70)**	**0.03**	**4.64 (1.80–14.95)**	**0.04**
Any alpha-7	2 (4.66)	2.79 (0.38–20.40)	0.19	3.70 (0.33–20.47)	0.37

*Overall there were 118 men with abnormal anal cytology at enrolment of which 45 had persistent abnormalities;

^#^n = 31 as 14 men were not on ART at enrolment,

^a^Adjusted Odds Ratio (aOR): Logistic Regression model included age, citizenship and having sex with other men;

^b^: CD4+ count >500 for all follow-up visits;

^c^: HIV-1 plasma viral load <40 copies/mL) for all follow-up visits;

^d^: Well controlled disease defined as on ART for >6 months, CD4+ >350 and undetectable PVL;

^e^: Alpha-7 includes: HPV 18, 39, 45 and 59;

^f^: Alpha-9 includes: HPV 16, 31, 33, 35, 52 and 58;

^h^: Persistent SILs were not associated with persistent HPV 16 or HPV 18 infections.

## Discussion

In this study of predominantly heterosexual men, we found that the prevalence of anal HPV infection was 39% and 26% of men with at least one anal HPV infection at enrolment had persistence 18 months later. The incidence of anal HPV was low at 0.79 per 100 person-years. By comparison, a study in China, also among heterosexual men, reported an incidence of 4.1 per 100 person-years, but the confidence interval of that study overlapped with ours. [24] Another recent study among HIV positive men who have sex with women in the US also reported low incidence for anal HPV 16 of 1.6 per 100 person years and this is despite a high HPV 16 prevalence of 60% reported at enrolment. [25] There are several potential reasons for the low incidence noted. Firstly, low incidence is expected in a largely heterosexual population, with higher incidence rates (range 21–46 per 100 person-years) reported in previous studies among MSM.[26, 27] Secondly, our participants had stable HIV disease since two thirds of the men were already on ART at enrolment with a median CD4+ count of 445 cells/μL and thus were likely able to mount a sufficient immune response to clear an infection which occurred soon after the first negative test. [25] In addition, the testing interval was at least one year, meaning that some incident HPV infections may have already been cleared by the time of the next assessment, leading to an underestimation of the true incidence rates. Despite the low number of new cases of anal HPV infection, persistence of anal HPV infection was high and comparable to rates that have been reported among other heterosexual MLHIV populations in Brazil, Italy, Mexico and US. [28, 29] This high proportion is a cause for concern as persistent anal HPV infection is necessary for progression to anal SIL.[3]

A third of participants in this cohort had persistent anal dysplasia, and a further quarter (27%) of MLHIV who had normal cytology at enrolment also developed dysplasia during follow-up. Despite this, there were no high-grade SIL cases at baseline or follow-up. The reason for this is unclear but could be related to the relatively short (18 months) follow-up period as progression of anal lesions may take a number of years.[30] However, the high proportion of new anal SIL accompanied by high rates of persistent anal SIL and comparatively low rates of regression suggests that these SIL persist for considerably long periods of time, and could become a cause for concern.[30] Despite these findings, we remain cautious about the need for a cytology screening programme for anal cancer among heterosexual MLHIV given that there were no high grade lesions and therefore more data, especially based on the number of cancers in this group, would be required for decision to be made. Based on the current understanding of the pathology of the natural history of HPV infection, ASCUS and LSIL lesions reflect the productive HPV viral replication from the excessive proliferation of all layers except the basal layer and thus their role as useful endpoints for anal cancer is unclear. It is rather the progression of untreated lesions to HSIL and the associated integration of the HPV genome into the host chromosomes, and the subsequent up regulation of E6 and E7 oncogene expression that would be more worrying as it will involve micro-invasive lesions. [31, 32]. Furthermore, given the lack of clear guidance of management of these lesions, screening might result in unnecessary psychological worries.

Lower CD4+ count had a strong inverse association with persistent anal HPV infection and persistent anal dysplasia. Quite likely, as in women living with HIV for cervical dysplasia, this is related to the role of cell-mediated immunity in the clearance of HPV infection or cells with pre-neoplastic changes.[7] ART status and duration on ART at enrolment were not significant predictors and this is in keeping with other previous reports.[33, 34] This is likely due to the fact that it is not the ART status *per se* that is important but other factors such early ART initiation, virological control and HIV disease status also influence the natural history of anal HPV infection and related disease.[13] This is aligned to our observation in this study that MLHIV with well controlled disease status had reduced odds of having persistent anal HPV infections. Therefore, initiatives to increase population HIV testing and immediate treatment initiation may be likely to provide benefit not only for HIV but also for prevention and control of HPV infection.

The strong association between anal HPV infection (both prevalent and persistent) and persistent anal dysplasia suggests that HPV vaccination could be used as an effective way of managing the future burden of anal dysplasia among MLHIV in high HIV prevalence settings such as South Africa. HPV vaccines can reduce the burden of HPV-related diseases, however the girls-only vaccination programme in South Africa might need to be extended to include boys if men are to be effectively protected. This is based on emerging evidence which suggest that the impact of a girls-only programme and herd immunity to men may be reduced by the high HIV prevalence in the country.[35] Moreover, MSM will not be protected by the herd immunity from a girls-only program.[36] More data on the cost effectiveness of including boys as well as effectiveness of a single dose HPV vaccine compared to the current two doses are required as they could support the case for including boys into a vaccination program.

Our study had a number of limitations. Conventional anal smears were used, which could have misclassified some cytological outcomes. Anal cytology screening is a relatively insensitive and nonspecific means of diagnosing anal HSIL.[37, 38] We did not have access to high resolution anoscopy and guided biopsy, the gold standard for pathological diagnosis. The small sample size for number of participants with persistent SILs also implies that the results for multivariate logistic regression should be interpreted with caution. In addition, since sexual behaviour was based on self-report, it is possible that some men were not willing to disclose whether they ever had sex with men. However, despite these limitations, we believe our study provides an important contribution to the epidemiology of anal HPV infection and dysplasia among a largely heterosexual MLHIV African population.

## Conclusions

In spite of a high prevalence of anal HPV, HIV-positive men have a low burden of anal HPV related disease. Men living with HIV face a significant burden of anal HPV infection and anal lesions. Effective, and possibly early, use of ART with immunological reconstitution and HIV virological control may contribute to the control of the HPV infection. HPV vaccination, if extended to boys, could also reduce this burden among both homosexual and heterosexual men in the future.

## Supporting information

S1 Table Performance of different HPV viral load cut-off points in predicting prevalent and persistent anal cytological abnormalities. *Values could not be calculated as all participants in that category had HPV VL above the cut-off point.
(DOC)

S2 Table Factors associated with persistent anal cytological abnormalities among heterosexual man only. (DOCX)

S1 File De-identified data set. (XLS)

S2 File Baseline CRF. (PDF)

S3 File Follow-up CRF. (PDF)

S4 File Calculation of incidence rate. (PDF)
