# Supplementary material for: Prevalent, persistent anal HPV infection and squamous intraepithelial lesions: Findings from a cohort of men living with HIV in South Africa
Source: PLoS One. 2019 Dec 5;14(12):e0225571. doi: 10.1371/journal.pone.0225571 (PMC6894774; doi:10.1371/journal.pone.0225571)
Supplement: S1 Table — *Values could not be calculated as all participants in that category had HPV VL above the cut-off point. (DOC) [file pone.0225571.s001.doc]

**Supplementary Table 1: Performance of different HPV viral load cut-off points in predicting prevalent and persistent anal SILs**

|  | **Prevalent SILs** | | | **Persistent SILs** | | |
| --- | --- | --- | --- | --- | --- | --- |
| **Description** | **Cut-off** | **Sensitivity (%)** | **Specificity (%)** | **Cut-off** | **Sensitivity (%)** | **Specificity (%)** |
| **HPV 16** | >3.2 | 33.3 | 87.5 | >0.0 | 100.0 | 0.0 |
| **viral load** | >3.6 | 25.0 | 87.5 | >3.2 | 14.3 | 100 |
| **(log_10_/10^6^ cells)** | >5.6 | 16.7 | 87.5 | -* | -* | -* |
|  | >5.9 | 8.3 | 87.5 | -* | -* | -* |
|  | >10.5 | 0.0 | 87.5 | -* | -* | -* |
|  | ROC area=0.58; 95% CI (0.37-0.79) | | | ROC area=0.57; 95% CI (0.43-0.63) | | |
| **Description** | **Cut-off** | **Sensitivity (%)** | **Specificity (%)** | **Cut-off** | **Sensitivity (%)** | **Specificity (%)** |
| **HPV 18** | >1.9 | 33.3 | 75.0 | >1.9 | 33.3 | 75.0 |
| **viral load** | >3.8 | 25.0 | 87.5 | >3.8 | 25.0 | 87.5 |
| **(log10/10^6^ cells)** | >4.9 | 25.0 | 100.0 | >4.9 | 25.0 | 100.0 |
|  | >6.9 | 16.7 | 100.0 | >6.9 | 16.7 | 100.0 |
|  | >7.9 | 8.3 | 100.0 | >7.9 | 8.3 | 100.0 |
|  | ROC area=0.56; 95% CI (0.35-0.77) | | | ROC area=0.56; 95% CI (0.32-0.79) | | |

*****Values calculated as all participants in that category had HPV VL above the cut-off point
