# Supplementary material for: Prevalent, persistent anal HPV infection and squamous intraepithelial lesions: Findings from a cohort of men living with HIV in South Africa
Source: PLoS One. 2019 Dec 5;14(12):e0225571. doi: 10.1371/journal.pone.0225571 (PMC6894774; doi:10.1371/journal.pone.0225571)
Supplement: S2 Table — (DOCX) [file pone.0225571.s002.docx]

**Supplementary Table 2: Factors associated with persistent anal cytological abnormalities among heterosexual man only***

| **Description** | | **N=41** | **Crude** | **P-value** | **Adjusted^a^** | **P-value** |
| --- | --- | --- | --- | --- | --- | --- |
|  | | **n (column %) or Median (IQR)** | **OR (95% CI)** |  | **aOR (95% CI)** |  |
| **ART status at enrolment** | |  |  |  |  |  |
|  | No ART | 12 (29.3) | 1 |  | 1 |  |
|  | ART | 29 (70.7) | 1.35 (0.63-2.91) | 0.45 | 1.23 (0.50-3.00) | 0.56 |
| **Duration on ART at enrolment (months)^#^** | |  |  |  |  |  |
|  | > 12 | 20 (71.4) | 1 |  | 1 |  |
|  | <12 | 8 (28.6) | **2.17 (1.07-9.87)** | **0.03** | **2.09 (1.04-7.23)** | **0.05** |
| **Enrolment CD4+ count (cells/μL)** | |  |  |  |  |  |
|  | >500 | 13 (34.2) | 1 |  | 1 |  |
|  | 351-500 | 12 (31.5) | 1.09 (0.45-2.68) | 0.85 | 1.13 (0.45-2.80) | 0.79 |
|  | 201-350 | 9 (23.7) | 1.42 (0.53-3.82) | 0.49 | 1.38 (0.51-3.75) | 0.52 |
|  | <200 | 4 (10.5) | 1.73 (0.45-6.67) | 0.43 | 1.62 (0.42-6.33) | 0.49 |
| **High stable CD4+ count^b^** | | 7 (17.1) | 0.70 (0.25-1.98) | 0.50 | 0.77 (0.30-1.96) | 0.58 |
| **Undetectable HIV-1 PVL (<40 copies/mL)** | | 17 (44.7) | 0.88 (0.42-1.84) | 0.73 | 0.96 (0.44-2.06) | 0.91 |
| **Sustained HIV virological control^c^** | | 28 (87.5) | 0.69 (0.26-1.84) | 0.96 | 0.70 (0.26-1.87) | 0.48 |
| **Disease control status at enrolment^d^** | |  |  |  |  |  |
|  | On ART, but poorly controlled | 12 (30.0) | 1 |  | 1 |  |
|  | ART naive | 16 (40.0) | 1.25 (0.53-2.93) | 0.61 | 1.08 (0.43-2.72) | 0.86 |
|  | Well controlled | 12 (30.0) | 1.48 (0.59-3.75) | 0.41 | 1.30 (0.49-3.48) | 0.60 |
| **Prevalent HPV infection at enrolment** | |  |  |  |  |  |
|  | Any-HPV | 13 (32.5) | 1.36 (0.62-2.98) | 0.44 | 1.24 (0.56-2.75) | 0.54 |
|  | Any HR-HPV | 12 (30.0) | **2.85 (1.20-6.79)** | **0.02** | **2.78 (1.14-6.75)** | **0.03** |
|  | Any alpha-7^e^ | 5 (12.5) | 1.91 (0.59-6.22) | 0.28 | 1.79 (0.54-5.87) | 0.34 |
|  | Any alpha-9^f^ | 6 (15.0) | **2.72 (1.08-8.65)** | **0.02** | **2.62 (1.11-8.52)** | **0.05** |
|  | HPV 16 | 3 (7.5) | 2.25 (0.48-10.52) | 0.30 | 2.11 (0.44-10.08) | 0.34 |
|  | HPV 18 | 2 (5.0) | 2.97 (0.40-21.84) | 0.28 | 3.15 (0.42-23.70) | 0.27 |
|  | HPV 45 | 3 (7.5) | 2.25 (0.48-10.52) | 0.30 | 1.86 (0.39-8.93) | 0.44 |
| **Persistent HPV infection^h^** | |  |  |  |  |  |
|  | Any-HPV | 4 (10.0) | **3.08 (1.07-12.96)** | **0.03** | **2.90 (1.06-12.39)** | **0.05** |
|  | Any HR-HPV | 2 (5.0) | **2.97 (1.04-21.84)** | **0.04** | **3.50 (1.46-26.39)** | **0.05** |
|  | Any alpha-7 | 1 (2.5) | 1.45 (0.13-16.42) | 0.77 | 1.58 (0.13-18.41) | 0.71 |
|  |  |  |  |  |  |  |

^*^Overall there were 120 men with abnormal anal cytology at enrolment of which 45 had persistent abnormalities; ^#^n=31 as 14 men were not on ART at enrolment, ^a^Adjusted Odds Ratio (aOR): Logistic Regression model included age and citizenship; ^b^: CD4+ count >500 for all follow-up visits; ^c^: HIV-1 plasma viral load <40 copies/mL) for all follow-up visits*;* ^d^: Well controlled disease defined as on ART for >6 months, CD4+ >350 and undetectable PVL; ^e^: Alpha-7 includes: HPV 18, 39, 45 and 59; ^f^: Alpha-9 includes: HPV 16, 31, 33, 35, 52 and 58; ^h^: Persistent SILs were not associated with persistent HPV 16 or HPV 18 infections.
