# Supplementary material for: Prevalent, persistent anal HPV infection and squamous intraepithelial lesions: Findings from a cohort of men living with HIV in South Africa
Source: PLoS One. 2019 Dec 5;14(12):e0225571. doi: 10.1371/journal.pone.0225571 (PMC6894774; doi:10.1371/journal.pone.0225571)
Supplement: S2 File — (PDF) [file pone.0225571.s004.pdf]

Case Report Form HIM: Enrolment visit

Participant ID	Visit Date	Staff Initials	Visit Code
□□□□-□	□□/□□□/□□□□<br>D D / M M/ Y Y Y Y	□□□	□□.□

INFORMED CONSENT FORM FOR PHOTOGRAPHY		
Has the participant consented for to have photographs of genital lesion to be taken.	Yes ☐	No ☐

1. SOCIO-DEMOGRAPHICS		
I'd like to ask you some questions of a personal nature. Please be as honest as you can in your responses. Remember that your responses are confidential and will not be shared with anyone else. This information will be used solely for research purposes.		
1.1 Where were you born?	Gauteng☐	North West Province☐
	KwaZulu-Natal☐	Mpumalanga☐
	Eastern Cape☐	Western Cape☐
	Free State☐	Northern Cape☐
	Limpopo ☐	
	Outside South Africa☐	→Specify____________
1.2 Which ethnic group do you belong to?	Black ☐	Coloured ☐
	White ☐	Indian ☐
	Other ☐	→Specify_____________ ____
1.3 What is your home language ?	isiZulu ☐	Ndebele ☐
	SeSotho ☐	Tshivenda ☐
	isiXhosa ☐	Xitsonga ☐
	SeTswana ☐	English ☐
→*Tick* ***ONLY*** *one option*	SePedi ☐	Afrikaans ☐
	SiSwati☐	
	Other ☐	→Specify____________
1.4 How long have you lived in Johannesburg?	<1 month☐	
	1-12months ☐	
	> 1 year ☐	→Years ☐☐

1.5 How many nights did you sleep outside of Johannesburg in the past 3 months? → ☐☐ number	
1.6 Are you planning to travel outside of Johannesburg in the next 12 months for more than a month at a time?	Yes ☐ No ☐
1.7.a What is your current marital status?	Single ☐ Divorced/ separated ☐ Married ☐ Widowed ☐
→1.7.b IF **MARRIED**, do you have more than one wife?	Yes ☐ →Number ☐☐ No ☐
1.8.a Do you have children?	Yes ☐ No ☐
→1.8.b IF **YES**, and number of children > 1 do all your children have the same mother do all your children have the same mother?	→ Number ☐☐ Yes ☐ No ☐
1.9 With whom do you live primarily?	Alone ☐ Partner ☐ Family ☐ Parents ☐ Sister ☐ Relatives ☐ Friend ☐ Shelter ☐ Other →Specify ________________
1.10What type of housing do you stay in?	Room in house/flat*(shared accommodation)* ☐ Hostel ☐ Flat ☐ Student Residence ☐ House ☐ Informal Dwelling ☐
1.11 What is the **highest** level of education you have completed?	No Schooling ☐ Incomplete secondary*(up to grade 11 or equivalent)* ☐ Primary ☐ Completed secondary*(Grade 12 or equivalent)* ☐ Graduate/ postgraduate ☐
1.12 What is your current employment status?	Unemployed ☐ Retired ☐ Employed full-time ☐ Other ☐ Employed part-time ☐ Specify↓ Self-employed ☐ ________________________
1.13 What is your main occupation (or last occupation if currently	Security guard ☐ Trader☐

unemployed)?<br><br>→*Tick **ONLY** one option*	Service*(waiter, cleaning)* ☐ Driver ☐<br>Builder/labourer ☐ Administrator/clerk ☐<br>Sales assistant/cashier ☐ Professional ☐ *(teacher, nurse, engineer, etc)*<br>Scholar/student ☐
1.14 Where do you usually seek health care?<br><br>→*Tick **ONLY** one option*	Primary health care facility ☐ Pharmacy ☐<br>Traditional healer ☐ Private doctor ☐
1.15 On a scale of 0 to 5 [where 0 is never and 5 is always] how often do you and your family go without eating food in an average month?	☐ 0 ☐ 1 ☐ 2 ☐ 3 ☐ 4 ☐ 5
1.16 On a scale of 0 to 5 [where 0 is easy and 5 is difficult] how easy or difficult would it be for you to find R100 for medicines if a person in your family became ill and needed treatment urgently?	☐ 0 ☐ 1 ☐ 2 ☐ 3 ☐ 4 ☐ 5
1.17 Do you personally own any of the following items?	Items / *Yes* / *No*<br>Working car ☐ ☐<br>Computer ☐ ☐<br>Fridge ☐ ☐<br>Television ☐ ☐<br>Radio ☐ ☐<br>Cell phone ☐ ☐
1.18 What was your monthly income **on average** in the last 3 months?	R ☐☐☐☐☐.☐☐

→Go to 2. ENVIRONMENT

**2. ENVIRONMENT**
Now I'd like to ask you some questions about your community and the place in which you live

2.1 In terms of violence, how safe do you consider your community/area/place in which you live?		Very safe ☐<br>Safe ☐<br>Unsafe ☐<br>Very unsafe ☐
2.2 How often have you heard about problems with crimes such as burglaries, hijackings, rapes, muggings or anything like that in the area that you live?	Never ☐	Once ☐<br>More than once ☐
2.3 How often have you heard about problems with violence directed against foreigners in the area that you live?	Never ☐	Once ☐<br>More than once ☐
2.4 Have you ever experienced any burglaries, hijackings, rapes, muggings, or violence against foreigners, directed at you?	Never ☐	Once ☐<br>More than once ☐
2.5 Have you ever spent time in prison for a crime?	Yes ☐	No ☐

→Go to 3. PARTICIPATION IN RESEARCH

3. PARTICIPATION IN RESEARCH

Now I'd like to ask you some questions about your participation in this study

3.1 Where did you hear about this study?

→*Tick* ***MULTIPLE RESPONSES*** *if applicable*

Source	Yes	No
Partner	☐	☐
Community health worker	☐	☐
SMS	☐	☐
Male friend	☐	☐
Radio	☐	☐
Poster/leaflet	☐	☐
Other	☐	☐

Specify Other ↓

____

3.2.a Have you told anyone about volunteering for this study? Yes ☐ No ☐

→3.2.b IF **YES**, whom did you tell and what was their reaction?

Person	Yes	No	Positive	Negative	Indifferent
Spouse/partner	☐	☐	☐	☐	☐
Parent	☐	☐	☐	☐	☐
Sibling	☐	☐	☐	☐	☐
Other relative	☐	☐	☐	☐	☐
Friends/neighbour	☐	☐	☐	☐	☐
Other health care worker	☐	☐	☐	☐	☐
Priest/church elder	☐	☐	☐	☐	☐
Traditional healer	☐	☐	☐	☐	☐

3.3 Have you ever taken part in any other trial for any medical products?

Yes ☐ →Date ☐☐/☐☐/☐☐☐☐

No ☐

→Go to 4.MEDICAL HISTORY

4. MEDICAL HISTORY

Now, I'd like to ask you some general questions about your health.

4.1 Have you ever suffered from any diseases of the:	Yes	No	Don't Know	→*IF YES give details:*
Heart	☐	☐	☐	
Lungs	☐	☐	☐	
Liver	☐	☐	☐	
Kidneys	☐	☐	☐	
Blood	☐	☐	☐	
Digestive system	☐	☐	☐	
Brain and nerves	☐	☐	☐	
Hormonal system	☐	☐	☐	
Skin	☐	☐	☐	
Autoimmune	☐	☐	☐	

4.2 Have you ever suffered from jaundice or hepatitis?	Yes ☐<br>↓details<br>____________________	No ☐
4.3 Have you ever had fits, faints, seizures or convulsions after the age of 5?	Yes ☐	No ☐
4.4 Have you ever had any psychiatric problems?	Yes ☐	No ☐
4.5 Have you or your family ever had any bleeding or clotting disorders?	Yes ☐	No ☐
4.6 Have you ever had a blood transfusion or received blood products?	Yes ☐	No ☐
4.7 Do you suffer from eczema or asthma?	Yes ☐	No ☐
4.8 Have you ever suffered from TB?	Yes ☐	No ☐
4.9 Have you ever suffered from shingles?	Yes ☐	No ☐

4.10 What is the WHO clinical stage?	I ☐<br>II ☐<br>III ☐<br>IV ☐
4.11 How would you rate your health overall in the past 12 MONTHS?	Excellent ☐ Fair ☐<br>Very good ☐ Poor (bad) ☐<br>Good ☐

4.12 In the last 12 MONTHS, did you suffer from any of the following health complaints:	**Yes**	**No**
Fever for > 1 month	☐	☐
Persistent cough for >1 month	☐	☐
Night sweats for > 1 month	☐	☐
Pain or difficulty with swallowing	☐	☐
Diarrhoea (i.e. >2 loose stools/day) for >1month	☐	☐
Persistent mouth or genital sores for > 1 month	☐	☐
Unintended loss of weight (> 10% body weight, i.e. >7 kg for an average man)	☐	☐
Skin rashes	☐	☐
Swollen glands	☐	☐
Fits	☐	☐

4.13 In the past 12 MONTHS, did a health care provider diagnose you with any of the following conditions:	**Yes**	**No**
TB	☐	☐
Pneumonia	☐	☐
Shingles	☐	☐
Oral thrush	☐	☐
Meningitis	☐	☐
Kaposi's Sarcoma	☐	☐

4.14 In the past 12 MONTHS, have you been admitted to hospital?	Yes ☐ → Specify______________<br>No ☐

4.15 What is your ART status?	**Status**<br><br>Never taken ARVs ☐ **Go to 4.20**<br><br>On treatment ☐ **Go to 4.16**<br><br>Past prophylaxis ☐ **Go to 4.20**	
4.16a First date on ART		☐☐/☐☐/☐☐☐☐ **(dd/mm/yyyy)**
4.16b For how long have you been on ART?		☐☐ years ☐☐ months
4.16c1 What is the ART code (Refer to the HIM-SA ATC code)<br><br>4.16c2 What is the therapy edge number?		☐☐☐☐☐☐☐<br><br>**(***Drop down menu eg* ***J05XX06)***<br><br>☐☐☐☐☐☐☐ **N/A** ☐
4.17 What is the line of treatment?	1 ☐<br><br>2 ☐<br><br>3 ☐	≥3 ☐<br><br>Missing ☐<br><br>Unknown ☐
4.18 How well do you adhere to your ART?	☐ None (< 10%)<br><br>☐ A few times (10-30%)<br><br>☐ About half the times (30-60%)	☐ Most of the times (60-90%)<br><br>☐ All (>90%)<br><br>☐ Unknown

4.19 What are some of the reason(s) that make you not take your medication? (*Tick all that apply*)	Reason Yes No<br>Toxicity ☐ ☐<br>Share with others ☐ ☐<br>Forgot ☐ ☐<br>Felt better ☐ ☐<br>Stock out ☐ ☐<br>Lost pills ☐ ☐	Reason Yes No<br>Missed appointment ☐ ☐<br>Ran out of Rx ☐ ☐<br>They taste badly -☐ ☐<br>Other ☐ ☐<br>Unknown ☐ ☐
4.20a Are you on any prescribed medications?		Yes No<br>☐ ☐<br>**If yes fill the Concomitant Log**
4.20b Are you on any other medication including traditional, home remedies or over the counter?		Yes No<br>☐ ☐
4.21a Do you know what was your lowest **(nadir)** CD4+ ?<br><br>4.21b When was the count and date of nadir CD4+ ?		Yes No<br>☐ ☐ **If yes go to 4.21b**<br>**If no go 5.**<br><br>CD+4 Count ☐☐☐<br>**Date ☐☐/☐☐☐/☐☐☐☐ (dd/mm/yyyy**

5. LIFESTYLE AND RISK BEHAVIOURS

Now I'm going to ask you some questions about your use of alcohol, tobacco and other drugs. I am going to ask you questions about your experiences of using these substances across your lifetime and more recently. While some of this information may be embarrassing or difficult to remember, please try and answer as truthfully as you can. Your responses are confidential and will only be recorded for research purposes.

5.1 Alcohol

	0	1	2	3	4
	**Never**	**Once a month or less**	**2 to 4 times a month**	**2 to 3 times a week**	**4 or more times a week**
5.1.1 How often do you have a drink containing alcohol?	☐	☐	☐	☐	☐

→*IF* ***0****, skip to* ***5.2 Smoking***

5.1.2 What kind of alcohol do you usually drink?

→*Tick* ***MULTIPLE RESPONSES*** *if applicable*

Type of alcohol	Yes	NO	Type of alcohol	Yes	NO
Beer/cider/alco-pop	☐	☐	Wine/champagne/port/sherry	☐	☐
Spirits	☐	☐	Home-brew/traditional beer	☐	☐
			Other Specify ______________↓		

	0	1	2	3	4
	**1 or 2**	**3 or 4**	**5 or 6**	**7 to 9**	**10 or more**
5.1.3 How many drinks containing alcohol do you have on a typical day when you are drinking?<br>***A drink is defined as….***<br>***1 can (330 ml) beer***<br>***1 small glass (140 ml) wine***<br>***1 shot spirits***	☐	☐	☐	☐	☐
	**Never**	**Less than monthly**	**Monthly**	**Weekly**	**Daily or almost daily**
5.1.4 How often do you have 6 or more drinks on one occasion?	☐	☐	☐	☐	☐

**SCORE**	=

→*IF **question 5.1.4** is **0**, skip to **5.2 Smoking***

↓*For respondents who have 6 or more drinks on one occasion*

	0	1	2	3	4
	**Never**	**Less than monthly**	**Monthly**	**Weekly**	**Daily or almost daily**
5.1.5 How often during the last 12 months have you found that you were not able to stop drinking once you started?	☐	☐	☐	☐	☐
5.1.6 How often during the last 12 months did you fail to do what was normally expected of you because of drinking?	☐	☐	☐	☐	☐
5.1.7 How often in the last 12 months have you needed a drink first thing in the morning to get yourself going after a heavy drinking session?	☐	☐	☐	☐	☐
5.1.8 How often in the past 12 months have you had a feeling of guilt or remorse after drinking?	☐	☐	☐	☐	☐
5.1.9 How often in the past 12 months have you been unable to remember what happened the night before because of your drinking?	☐	☐	☐	☐	☐
	**No**		**Yes, but not in the last year**		**Yes, during the last year**
5.1.10 Have you or someone else been injured as a result of your drinking?	☐		☐		☐
5.1.11 Has a relative, friend, doctor or other health care worker been concerned about your drinking or suggested that you cut down?	☐		☐		☐

	SCORE	=

5.2 Smoking

5.2.1.a Do you currently smoke cigarettes? Yes ☐ →Specify how many per day on average ☐☐
No ☐

→5.2.1.b IF **NO**, have you ever smoked cigarettes? Yes ☐ →Date stopped ☐☐/☐☐☐/☐☐☐☐
No ☐

5.3 Substance use

Now, I am going to ask you some questions about your experience of using substances other than those prescribed by a doctor for medical reasons. These substances can be smoked, swallowed, inhaled, injected or taken in the form of pills. Please be assured that the information on substance use will be treated as strictly confidential. We will use this information to assist us to identify any health needs you may have.

5.3.1 In your lifetime, which of the following substances have you ever used other than for prescribed medical reasons?	**Never**	**Once**	**More than once**
Cannabis (dagga, marijuana, hash)	☐	☐	☐
Inhalants (glue, petrol, paint thinners)	☐	☐	☐
Cocaine (coke, crack, freebase)	☐	☐	☐
Amphetamines (tik, khat, Ritalin)	☐	☐	☐
Sedatives or sleeping pills (mandrax, valium, dormicum)	☐	☐	☐
Opiates (heroin, opium, morphine, codeine)	☐	☐	☐
Hallucinogens (ecstasy, LSD)	☐	☐	☐
GHB (gamma hydroxyl butyrate)	☐	☐	☐
Others<br>↓ Specify______________________________	☐	☐	☐

→*IF* ***NEVER*** *to* ***all*** *items above skip to* ***5.4 Violence***

These questions relate to your use of substances over the PAST 12 MONTHS

	**0**	**2**	**3**	**4**	**6**
5.3.2 In the past 12 months, how often have you used the substances you mentioned?	**Never**	**Once or twice**	**Monthly**	**Weekly**	**Daily or almost daily**
Cannabis	☐	☐	☐	☐	☐
Inhalants	☐	☐	☐	☐	☐
Cocaine	☐	☐	☐	☐	☐
Amphetamines	☐	☐	☐	☐	☐
Sedatives incl. Mandrax	☐	☐	☐	☐	☐
Opiates	☐	☐	☐	☐	☐
Hallucinogens	☐	☐	☐	☐	☐
GHB	☐	☐	☐	☐	☐
Other ↓Specify ____________________	☐	☐	☐	☐	☐
				**SCORE**	=

→IF ***NEVER*** *to all items above, is* ***0,*** *skip to* ***5.4 Violence***

↓*For respondents who report substance use in PAST 12 MONTHS*

	0	2	3	4	6
	**Never**	**Once a month or less**	**2 to 4 times a month.**	**2 to 3 times a week.**	**4 or more times a week.**
5.3.3 Do you use more than one type of drug on the same occasion?	☐	☐	☐	☐	☐
	**Never**	**Less than monthly**	**Monthly**	**Weekly**	**Daily or almost daily**
5.3.4 How often in the past 12 months have you had a strong desire or urge to use drugs that you found hard to resist?	☐	☐	☐	☐	☐
5.3.5 How often in the last 12 months has it happened that you have not been able to stop taking drugs once you started?	☐	☐	☐	☐	☐
5.3.6 How often in the last 12 months did you fail to do what was normally expected of you because of drug use?	☐	☐	☐	☐	☐
5.3.7 How often in the last 12 months have you had to take drugs first thing in the morning to get yourself going after a heavy drug use session the day before?	☐	☐	☐	☐	☐
5.3.8 How often in the past 12 months have you had a feeling of guilt or remorse after using drugs?	☐	☐	☐	☐	☐
				**SCORE**	=

↓*For respondents who report substance use in PAST 12 MONTHS*

	**Yes, during the last 12 months**	**Yes, but not in the last 12 months**	**No**
5.3.9 Have you become involved in fights while under the influence of drugs?	☐	☐	☐
5.3.10 Have you engaged in sex for money, goods or drugs in order to obtain drugs?	☐	☐	☐
5.3.11 Have you engaged in illegal activities in order to obtain drugs?	☐	☐	☐
5.3.12 Have you been arrested for possession of illegal drugs?	☐	☐	☐
5.3.13 Have you ever had blackouts or flashbacks as a result of using drugs?	☐	☐	☐
5.3.14 Have you ever experienced withdrawal symptoms (felt sick) when you stopped taking drugs?	☐	☐	☐
5.3.15 Have you ever had medical problems as a result of your drug use (e.g. memory loss, fits, bleeding or hepatitis)?	☐	☐	☐
5.3.16 Have you or someone else been mentally or physically hurt because you used drugs?	☐	☐	☐
5.3.17 Has a relative, friend, doctor or other health care worker been concerned about your drug use or suggested that you should stop?	☐	☐	☐
5.3.18.a Have you ever injected drugs?	☐	☐	☐
→5.3.18.bIF **YES**, how often did you inject drugs in the past 12 months?		Once weekly or less ☐<br>More than once per week ☐	

**5.4 Violence**			
You are doing really well and we are about half-way of the way through the interview. The information you have given us is very important.<br><br>*There are now a few more questions about your experiences growing up, and your relationships with others. Some men find these questions hard to talk about, others find it easy. Remember that everything you share here will only be used for research purposes and will be kept strictly secret. We have not written down your name anywhere and what you say in this interview cannot be linked to you in any way, but it WILL help us a lot in understanding the lives of men in South Africa.*			
	**Never**	**Once**	**More than once**
5.4.1 While you were growing up, how often did someone in your household push, grab, shove, throw something, slap, hit, kick or punch you?	☐	☐	☐
5.4.2 While you were growing up, how often did your parents or the people who raised you push, grab, shove, throw something, slap, hit, kick or punch each other?	☐	☐	☐

→Go to 6. SEXUAL BEHAVIOUR AND PRACTICES

**6. SEXUAL BEHAVIOUR AND PRACTICES**		
I'd now like to ask you about your sexual partners and practices. While some of this information may be embarrassing or difficult to remember, please try and answer as truthfully as you can.		
6.1.1 Have you ever had sexual intercourse?<br>*(By this I mean penetrative sex where the penis enters the vagina, anus or mouth)*	Yes ☐	No ☐<br>→*IF **NO**, participant is not eligible*
6.1.2 At what age did you first have sex?	Years ☐☐	
6.1.3 The first time you had sex, were you forced?	Yes ☐	No ☐
6.1.4 How many sexual partners have you had in your lifetime?	→*IF 0 partners participant is not eligible*	☐☐☐ partners
6.1.5.a How many sexual partners have you had in the past 3 months?	→*IF 0 partners participant is not eligible*	☐☐ partners
→6.1.5.bIF >**1 partner**, did you have a relationship with more than one person at the same time during the last 3 months?	Yes ☐	No ☐
6.1.6.a Some men have sex with other men. Have you ever had sex with another man?	Yes ☐	No ☐
→6.1.6.b IF **YES,** at what age did you first have sex with another man?	Years ☐☐	
→6.1.6.c The first time you had sex with a man did you have sex against your will?	Yes ☐	No ☐
6.1.7 How do you identify yourself?	Heterosexual/Straight ☐<br>Bisexual ☐<br>Homosexual/Gay ☐<br>Other ☐<br>→ Specify ____________	

Thanks for answering those. The information you have given us is very important. There are now a few more questions about things you may ***have done with women*** *whether they were partners or girlfriends or other women. These next questions are asking about the whole of your life, including when you were a boy. Please feel free in answering these questions, we really want to learn more about things men do. Remember that what your answer here cannot be linked to you in any way.*

	**Never**	**Once**	**More than once**
6.1.8 How many times have you slept with a woman or girl when she didn't consent to sex or after you forced her?	☐	☐	☐
6.1.9 How many times have you slept with a woman or girl when she was too drunk to say whether she wanted it or not?	☐	☐	☐
6.1.10 Have you ever done anything sexual with a boy or man when he didn't consent or you forced him?	☐	☐	☐
6.1.11 Have you ever had sex with someone because they threatened you?	☐	☐	☐

6.2 Partners

I'd like to know more about your most recent sexual partner in the last 3 months up to a maximum of 3 partners ***as indicated in Question 6.15a*** *starting with your most recent sexual partner.*

For these questions, sex is defined as any vaginal or anal sex that is where a man inserts his penis into the vagina or anus. A sexual partner is someone with whom you have had sex either regularly or only once.

6.2.1 Most recent partner

6.2.1.a How old is this person?	Years ☐☐	
6.2.1.b Is this person male or female?	Female ☐	Male ☐
6.2.1.c How would you characterise your relationship with this person?<br>***Main partner*** *=person you regularly have sex with AND married to/living with*<br>***Regular partner*** *= person you regularly have sex with but not married/not living together*<br>***Occasional partner*** *= person you had sex with once or twice*	Main partner ☐<br>Occasional partner ☐	Regular partner ☐

6.2.1.d Is this person a new partner, i.e. someone that you had sex with for the first time in the past 3 months?	Yes ☐	No ☐
6.2.1.e Is this partner a recent immigrant to South Africa (i.e. arrived within the last 3 years)?	Yes ☐<br>Don't know ☐	No ☐
6.2.1.f Has this person ever tested positive for HIV?	Yes ☐<br>Don't know ☐	No ☐
6.2.1.g How long did you know this person before you first had sex with them?	Minutes ☐☐<br>Hours ☐☐<br>Weeks ☐☐	Months ☐☐<br>Years ☐☐
6.2.1.h During the last 3 months, how many times did you have sex with this partner?	No. of times ☐☐☐	
6.2.1.i How often did you use a condom when you had sex?	Always ☐<br>>/= to half the time ☐	< half the time☐<br>Never ☐
6.2.1.j Did you drink alcohol the last time that you had sex with this partner (either during sex or up to two hours before you had sex)?	Yes ☐	No ☐
6.2.1.k Did this partner give you or receive from you money, drugs, food or a place to stay in exchange for sex the last time that you had sex?	Yes ☐	No ☐

6.2.1.l What type of sex did you have with this person?

→ ***MULTIPLE RESPONSES*** *possible*

Type of sex	Yes	No
Oral received	☐	☐
Oral performed	☐	☐
Vaginal	☐	☐
Receptive insertion of digits in anus	☐	☐
Receptive insertion of other objects in anus	☐	☐
Anal receptive	☐	☐
Anal insertive	☐	☐
Rimming received	☐	☐
Rimming performed	☐	☐

6.2.1.m Do you know or suspect that this person has other sexual partners?	Yes ☐	No ☐

**6.2.2 Second most recent partner in past 3 months**		
6.2.2.a How old is this person?	Years ☐☐	
6.2.2.b Is this person male or female?	Female ☐	Male ☐
6.2.2.c How would you characterise your relationship with this person?<br>***Main partner*** *=person you regularly have sex with AND married to/living with*<br>***Regular partner*** *= person you regularly have sex with but not married/not living together*<br>***Occasional partner*** *= person you had sex with once or twice*	Main partner ☐<br>Occasional partner ☐	Regular partner ☐
6.2.2.d Is this person a new partner, i.e. someone that you had sex with for the first time in the past 3 months?	Yes ☐	No ☐
6.2.2.e Is this partner a recent immigrant to South Africa (i.e. arrived within the last 3 years)?	Yes ☐<br>Don't know ☐	No ☐
6.2.2.f Has this person ever tested positive for HIV?	Yes ☐<br>Don't know ☐	No ☐
6.2.2.g How long did you know this person before you first had sex with them?	Minutes ☐☐<br>Hours ☐☐<br>Weeks ☐☐	Months ☐☐<br>Years ☐☐
6.2.2.h During the last 3 months, how many times did you have sex with this partner?	No. of times ☐☐	
6.2.2.i How often did you use a condom when you had sex?	Always ☐<br>>/= to half the time ☐	< half the time ☐<br>Never ☐
6.2.2.j Did you drink alcohol the last time that you had sex with this partner – either during sex or up to two hours before you had sex?	Yes ☐	No ☐

6.2.2.k Did this partner give you or receive from you money, drugs, food or a place to stay in exchange for sex the last time that you had sex?	Yes ☐	No ☐
6.2.2.l What type of sex did you have with this person?<br><br>→***MULTIPLE RESPONSES*** *possible*	Type of sex Yes No<br>Oral received ☐ ☐<br>Oral performed ☐ ☐<br>Vaginal ☐ ☐<br>Anal receptive ☐ ☐<br>Anal insertive ☐ ☐	
6.2.2.m Do you know or suspect that this person has other sexual partners?	Yes ☐	No ☐
**6.2.3 Third most recent partner in past 3 months**		
6.2.3.a How old is this person?	Years ☐☐	
6.2.3.b Is this person male or female?	Female ☐	Male ☐

6.2.3.c How would you characterise your relationship with this person?<br>***Main partner*** *=person you regularly have sex with AND married to/living with*<br>***Regular partner*** *= person you regularly have sex with but not married/not living together*<br>***Occasional partner*** *= person you had sex with once or twice*	Main partner ☐<br>Occasional partner ☐	Regular partner ☐
6.2.3.d Is this person a new partner, i.e. someone that you had sex with for the first time in the past 3 months?	Yes ☐	No ☐
6.2.3.e Is this partner a recent immigrant to South Africa (i.e. arrived within the last 3 years)?	Yes ☐<br>Don't know ☐	No ☐
6.2.3.f Has this person ever tested positive for HIV?	Yes ☐	No ☐

	Don't know ☐	
6.2.3.g How long did you know this person before you first had sex with them?	Minutes ☐☐<br>Hours ☐☐<br>Weeks ☐☐	Months ☐☐<br>Years ☐☐
6.2.3.h During the last 3 months, how many times did you have sex with this partner?	No. of times ☐☐	
6.2.3.i How often did you use a condom when you had sex?	Always ☐<br>>/= to half the time ☐	< half the time☐<br>Never ☐
6.2.3.j Did you drink alcohol the last time that you had sex with this partner – either during sex or up to two hours before you had sex?	Yes ☐	No ☐
6.2.3.k Did this partner give you or receive from you money, drugs, food or a place to stay in exchange for sex the last time that you had sex?	Yes ☐	No ☐
6.2.3.l What type of sex did you have with this person?<br><br>→***MULTIPLERESPONSES*** *possible*	Type of sex Yes No<br>Oral received ☐ ☐<br>Oral performed ☐ ☐<br>Anal insertive ☐ ☐<br>Anal receptive ☐ ☐<br>Vaginal ☐ ☐	
6.2.3.m Do you know or suspect that this person has other sexual partners?	Yes ☐	No ☐

→Go to 7. GENITAL HYGIENE PRACTICES

7. GENITAL HYGIENE PRACTICES

Now I'm going to ask you some questions about your personal hygiene practices. While some of these questions may be embarrassing or difficult to answer, please try and answer as truthfully as you can.

7.1.a Are you circumcised?

Yes ☐ → Age of circumcision ☐☐

No ☐

→7.1.b Where were you circumcised?

Traditional initiation school ☐ Other religious setting ☐

Health care facility ☐ Don't know ☐

7.2.a Do you clean your penis before and/or after you have sex?

Yes, before sex ☐

Yes, before and after sex ☐

Yes, after sex ☐

No ☐ →*IF* ***NO****, go to 8.4.a*

→7.2.b IF **YES**, what do you use to clean your penis before and/or after sex?

→*Tick* ***MULTIPLE RESPONSES*** *if applicable*

Item	Yes	No
Water	☐	☐
Water and soap	☐	☐
Imbiza/herbs	☐	☐
Cloth/towel to wipe/apply products	☐	☐
Other household products	☐	☐

7.3 How often do you clean your penis at the time of sex?

Always ☐

More than half of sex acts ☐

Less than half of sex acts ☐

7.4.a Did you use any substances to enhance your sexual performance?

Yes ☐ No ☐

→7.4.bIF **YES,** what products did you use to enhance your sexual performance?

(Substances: Imbiza/muti/herbs/powders)

**Substances**	**Yes**	**No**
Taken orally	☐	☐
Applied to penis	☐	☐
Pills	☐	☐
Other	☐	☐

Other specify__________

7.5 Do you know whether your sexual partner/s use products other than water to clean or tighten his/her genital area before and/or after sex?	Yes ☐<br>Don't know ☐	No ☐

→Go to 8.

8. SEXUAL HEALTH HISTORY

8.1 Have you ever had a sexually transmitted infection? Yes ☐ No ☐ Don't know ☐

8.2 In the last 3 months, has a health care provider other than someone from the study, diagnosed you with or treated you for any of the following sexually transmitted or genital infections:

		Yes	No	Don't know
	Burning urine or discharge from the penis	☐	☐	☐
	Genital ulcer disease (including genital herpes)	☐	☐	☐
	Swollen glands in the groin (Bubo)	☐	☐	☐
	Pain or swelling of testicles	☐	☐	☐
	Genital warts	☐	☐	☐
NA ☐	For **MSM**: Pain or discharge from the rectum	☐	☐	☐
	Syphilis (by serological test)	☐	☐	☐

8.3 Were you diagnosed with a sexually transmitted infection but don't know the name or it is not listed above? Yes ☐ → Details ________ No ☐ Don't know ☐

8.4 Were you given treatment because your partner had a sexually transmitted infection? Yes ☐ No ☐ Don't know ☐

8.5 Have you sought care from anywhere other than a government clinic in the past 12 months? Yes ☐ → Details ________ No ☐ → Go to Question 8.6

8.6 IF you received treatment, did you:

→8.6.a Complete your treatment as prescribed? Yes ☐ No ☐

→8.6.b Have sex while taking treatment? Yes ☐ No ☐

→8.6.cIF **you had sex while taking treatment,** did you use a condom for every sex act?

Yes ☐ No ☐

→8.6.d Were all your partners treated? Yes ☐ No ☐ Don't know ☐ Not applicable ☐

→8.6.e Do you still have any genital symptoms? Yes ☐ No ☐

Not applicable ☐

8.7 In the past 7 days, have you experienced:	Yes	No	Don’t Know
Burning urine or discharge from the penis	☐	☐	☐
Blisters or sores in the genital area	☐	☐	☐
Swollen glands in the groin (Bubo)	☐	☐	☐
Pain or swelling of testicles	☐	☐	☐
Genital warts	☐	☐	☐
NA ☐ For **MSM**: Pain or discharge from the rectum	☐	☐	☐
8.8 In the past 7 days have you been given treatment because your partner has an STI	☐	☐	☐

→Go to 9. PHYSICAL EXAMINATION

9. PHYSICAL EXAMINATION	
9.1 Body Temperature	☐☐.☐ °C
9.2 Height	☐.☐☐ meters
9.3 Weight	☐☐☐ . ☐ kilograms
9.4 Heart Rate	☐☐☐ per minute
9.5 Blood Pressure	Systolic: ☐☐☐ mmHg Diastolic: ☐☐☐ mmHg

**9.6 Body system or Part Observations** (only if required)	**Not Examined**	**Normal**	**Abnormal**	***→IF ABNORMAL, details below:***
a. Skin and mucous membranes	☐	☐	☐	
b. Head and neck	☐	☐	☐	
c. Cardiovascular	☐	☐	☐	
d. Respiratory	☐	☐	☐	
e. Gastrointestinal (abdominal & liver)	☐	☐	☐	
f. Neurological	☐	☐	☐	
g. Genital	☐	☐	☐	

**9.7 Genital Examination Observations**	**Not Examined**	**No**	**Yes**	***→IF YES details below:***	
a. Is participant circumcised?	☐	☐	☐	Partial ☐	Full ☐
b. Inguinal lymphadenopathy?	☐	☐	☐	Single ☐	Multiple ☐<br>Bubo ☐
c. Balanitis?	☐	☐	☐		
d. Discharge?	☐	☐	☐	Meatal ☐	Subpreputial ☐
**9.7 General examinations**	**Not examined**	**No**	**Yes**	**If yes give details**	

e.	Ulcers?	☐	☐	☐	
	IF **YES**, →				Ulcer Number: Single ☐ Multiple ☐
	→				Ulcer Location: Prepuce ☐ Glans ☐ Shaft ☐ Inguinal ☐ Buttocks ☐ Perianal ☐ Other ☐
					→ Ulcer Pain: Yes ☐ No ☐
					→ Ulcer Bleeding: Yes ☐ No ☐
					**IF YES** and **Consented to photography-Take a photo**
f.	Warts?	☐	☐	☐	Single ☐ Multiple ☐<br>Location:________________________<br>**IF YES** and **Consented to photography-Take a photo**
g.	Peri-anal abnormalities?	☐	☐	☐	Details.________________________<br>**IF YES** and **Consented to photography-Take a photo**
h.	Other genital abnormality or deformity?	☐	☐	☐	Details,________________________<br>**IF YES** and **Consented to photography-Take a photo**

→Go to 10. LABORATORY SAMPLE COLLECTION

**10. Samples collected**	
**Blood samples**	
HIV PVL	Yes☐ No ☐
CD4+ cell count	Yes☐ No ☐
HBsAntigen	Yes☐ No ☐
Syphilis serology:	
RPR	Yes☐ No ☐
TPHA	Yes☐ No ☐
HSV – 2 serology	Yes☐ No ☐
HPV serology	Yes☐ No ☐
**Urine samples**	
C. trachomatis PCR	Yes☐ No ☐
N. gonorrhoeae PCR	Yes☐ No ☐
T. vaginalis PCR	Yes☐ No ☐
M. genitalium PCR	Yes☐ No ☐
**Genital samples**	
HPV genital swab	Yes☐ No ☐
HPV anal canal swab	Yes☐ No ☐
Anal cytology swab	Yes☐ No ☐
**Oral samples**	
oral rinse sample	Yes☐ No ☐
oral HPV swab	Yes☐ No ☐

**11. DIAGNOSIS AND TREATMENT**		
*IF REQUIRED, please provide participant with **script for treatment** to be collected at the pharmacy and **partner notification slips.***		
11.1 Was the participant treated presumptively for any of the following at this visit?	**Yes**	**No**
Urethritis syndrome	☐	☐
Genital ulcer syndrome	☐	☐
Scrotal swelling	☐	☐
Balanitis / Balanoposthitis	☐	☐
Bubo	☐	☐
Molluscumcontagiosum	☐	☐
Genital Warts	☐	☐
Pubic lice	☐	☐
	**If Yes to any complete the concomitant Log**	
11.2.a Did the participant require treatment for ANY OTHER CONDITION?	Yes ☐<br>**If yes to any complete the concomitant Log**	No ☐
→11.2.b IF **YES**, provide details		
