# Supplementary material for: Prevalent, persistent anal HPV infection and squamous intraepithelial lesions: Findings from a cohort of men living with HIV in South Africa
Source: PLoS One. 2019 Dec 5;14(12):e0225571. doi: 10.1371/journal.pone.0225571 (PMC6894774; doi:10.1371/journal.pone.0225571)
Supplement: S3 File — (PDF) [file pone.0225571.s005.pdf]

Case Report Form **HIM: Follow-up Visit**

Participant ID	Visit Date	Visit Code	Staff Initials
□□□□-□	□□/□□/□□□□<br>D D / M M/ Y Y Y Y	□□.□	□□□

1. MEDICAL HISTORY

I'd like to ask you some general questions about your health. Since your last visit.

1.1 Have you suffered from any diseases of the following, in the last 6 months?	Yes	No	***→IF YES give details and complete the SAE log:***
Heart	☐	☐	
Lungs	☐	☐	
Liver	☐	☐	
Kidneys	☐	☐	
Blood	☐	☐	
Digestive system	☐	☐	
Brain and nerves	☐	☐	
Hormonal system	☐	☐	
Skin	☐	☐	
Autoimmune	☐	☐	

1.2 Have you suffered from jaundice or hepatitis in the last 6 months?	Yes ☐<br>↓details<br>____________________	No ☐
1.3 Have you had fits, faints, seizures or convulsions in the last 6 months?	Yes ☐	No ☐
1.4 Have you had any psychiatric problems in the last 6 months?	Yes ☐	No ☐
1.5 Have you or your family ever had any bleeding or clotting disorders?	Yes ☐	No ☐
1.6 Have you had a blood transfusion or received blood products in the last 6 months?	Yes ☐	No ☐
1.7 Do you suffer from eczema or asthma?	Yes ☐	No ☐

1.8 WHO clinical stage	I ☐<br>II ☐<br>III ☐<br>IV ☐		
1.9 How would you rate your health overall in the past 6 MONTHS?	Excellent ☐<br>Very good ☐<br>Good ☐	Fair ☐<br>Poor (bad) ☐	

1.10 In the last 6 MONTHS, did you suffer from any of the following health complaints:	**Yes**	**No**
Fever for > 1 month	☐	☐
Persistent cough for >1 month	☐	☐
Night sweats for > 1 month	☐	☐
Pain or difficulty with swallowing	☐	☐
Diarrhoea (i.e. >2 loose stools/day) for >1month	☐	☐
Persistent mouth or genital sores for > 1 month	☐	☐
Unintended loss of weight (> 10% body weight, i.e. >7 kg for an average man)	☐	☐
Skin rashes	☐	☐
Swollen glands	☐	☐
Fits	☐	☐

1.11 In the past 6 MONTHS, did a health care provider diagnose you with any of the following conditions:	**Yes**	**No**
TB	☐	☐
Pneumonia	☐	☐
Shingles	☐	☐
Oral thrush	☐	☐
Meningitis	☐	☐
Kaposi's Sarcoma	☐	☐

1.12 In the past 6 MONTHS, have you been admitted to hospital?	Yes ☐<br>No ☐	→ **Specify and fill the AE log**______________

1.13 What is your ART status?	**Status**<br><br>Never taken ARVs ☐ **Go to 1.18**<br><br>On treatment ☐ **Go to 1.14**<br><br>Past prophylaxis ☐ **Go to 1.18**	
1.14a First date on ART		☐☐/☐☐/☐☐☐☐ **(dd/mm/yyyy)**
1.14b For how long have you been on ART?		☐☐ years ☐☐ months
1.14c1 What is the ART code (Refer to the HIM-SA ATC code)<br><br>1.14c2 What is the therapy edge number?		☐☐☐☐☐☐☐<br><br>**(***Drop down menu eg* ***J05XX06)***<br><br>☐☐☐☐☐☐☐ **N/A** ☐
1.15 What is the line of treatment?	1 ☐<br><br>2 ☐<br><br>3 ☐	≥3 ☐<br><br>Missing ☐<br><br>Unknown ☐
1.16 How well do you adhere to your ART?	☐ None (< 10%)<br><br>☐ A few times (10-30%)<br><br>☐ About half the times (30-60%)	☐ Most of the times (60-90%)<br><br>☐ All (>90%)<br><br>☐ Unknown

1.17 What are some of the reason(s) that make you not take your medication? (*Tick all that apply*)	Reason Yes No<br>Toxicity ☐ ☐<br>Share with others ☐ ☐<br>Forgot ☐ ☐<br>Felt better ☐ ☐<br>Stock out ☐ ☐<br>Lost pills ☐ ☐	Reason Yes No<br>Missed appointment ☐ ☐<br>Ran out of Rx ☐ ☐<br>They taste badly ☐ ☐<br>Other ☐ ☐<br>Unknown ☐ ☐
1.18a Are you on any prescribed medications?		Yes No<br>☐ ☐<br>**If yes fill the Concomitant Log**
1.18b Are you on any other medication including traditional, home remedies or over the counter?		Yes No<br>☐ ☐
1.19a Do you know what was your lowest **(nadir**) CD4+ ?<br><br>1.19b When was the count and date of nadir CD4+ ?		Yes No<br>☐ ☐ **If yes go to 1.19b**<br>**If no go to 2.**<br>CD+4 Count ☐☐☐<br>**Date ☐☐/☐☐☐/☐☐☐☐ (dd/mm/yyyy**

2. LIFESTYLE AND RISK BEHAVIOURS

Now I'm going to ask you some questions about your use of alcohol, tobacco and other drugs SINCE YOUR LAST VISIT. I am going to ask you questions about your experiences of using these substances across your lifetime and more recently. While some of this information may be embarrassing or difficult to remember, please try and answer as truthfully as you can. Your responses are confidential and will only be recorded for research purposes.

2.1 Alcohol

	0	1	2	3	4
	**Never**	**Once a month or less**	**2 to 4 times a month**	**2 to 3 times a week**	**4 or more times a week**
2.1.1 How often do you have a drink containing alcohol?	☐	☐	☐	☐	☐

→*IF* ***0****, skip to 2****.2 Smoking***

2.1.2 What kind of alcohol do you usually drink?

→*Tick* ***MULTIPLE RESPONSES*** *if applicable*

Beer/cider/alco-pop ☐ Wine/champagne/port/sherry ☐

Spirits ☐ Home-brew/traditional beer ☐

Specify spirits____________↓

	0	1	2	3	4
	**1 or 2**	**3 or 4**	**5 or 6**	**7 to 9**	**10 or more**
2.1.3 How many drinks containing alcohol do you have on a typical day when you are drinking?<br>***A drink is defined as....***<br>***1 can (330 ml) beer***<br>***1 small glass (140 ml) wine***<br>***1 shot spirits***	☐	☐	☐	☐	☐
	**Never**	**Less than monthly**	**Monthly**	**Weekly**	**Daily or almost daily**
2.1.4 How often do you have 6 or more drinks on one occasion?	☐	☐	☐	☐	☐

**SCORE**	=

→*IF **question 2.1.4** is **0**, skip to **2.2 Smoking***

↓*For respondents who have 6 or more drinks on one occasion*

	0	1	2	3	4
	**Never**	**Less than monthly**	**Monthly**	**Weekly**	**Daily or almost daily**
2.1.5 How often during the last 12 months have you found that you were not able to stop drinking once you started?	☐	☐	☐	☐	☐
2.1.6 How often during the last 12 months did you fail to do what was normally expected of you because of drinking?	☐	☐	☐	☐	☐
2.1.7 How often in the last 12 months have you needed a drink first thing in the morning to get yourself going after a heavy drinking session?	☐	☐	☐	☐	☐
2.1.8 How often in the past 12 months have you had a feeling of guilt or remorse after drinking?	☐	☐	☐	☐	☐
2.1.9 How often in the past 12 months have you been unable to remember what happened the night before because of your drinking?	☐	☐	☐	☐	☐
	**No**		**Yes, but not in the last year**		**Yes, during the last year**
2.1.10 Have you or someone else been injured as a result of your drinking?	☐		☐		☐
2.1.11 Has a relative, friend, doctor or other health care worker been concerned about your drinking or suggested that you cut down?	☐		☐		☐

**SCORE**	=

**2.2 Smoking**		
2.2.1.a Do you currently smoke cigarettes?	Yes ☐<br>No ☐	→Specify how many per day on average ☐☐

**2.3 Substance use**
Now, I am going to ask you some questions about your experience of using substances other than those prescribed by a doctor for medical reasons. These substances can be smoked, swallowed, inhaled, injected or taken in the form of pills. Please be assured that the information on substance use will be treated as strictly confidential. We will use this information to assist us to identify any health needs you may have.

These questions relate to your use of substances over the PAST 6 MONTHS

	**0**	**2**	**3**	**4**	**6**
2.3.1 In the past 6 months, how often have you used these substances?	**Never**	**Once or twice**	**Monthly**	**Weekly**	**Daily or almost daily**
Cannabis	☐	☐	☐	☐	☐
Inhalants	☐	☐	☐	☐	☐
Cocaine	☐	☐	☐	☐	☐
Amphetamines	☐	☐	☐	☐	☐
Sedatives incl. Mandrax	☐	☐	☐	☐	☐
Opiates	☐	☐	☐	☐	☐
Hallucinogens	☐	☐	☐	☐	☐
GHB	☐	☐	☐	☐	☐
Other<br>↓Specify<br>________________	☐	☐	☐	☐	☐
				**SCORE**	=

→IF ***NEVER*** *to all items above, is* ***0,*** *skip to* ***2.4 Violence***

↓*For respondents who report substance use in PAST 6 MONTHS*

	0	2	3	4	6
	**Never**	**Once a month or less**	**2 to 4 times a month.**	**2 to 3 times a week.**	**4 or more times a week.**
2.3.2 Do you use more than one type of drug on the same occasion?	☐	☐	☐	☐	☐
	**Never**	**Less than monthly**	**Monthly**	**Weekly**	**Daily or almost daily**
2.3.3 How often in the past 6 months have you had a strong desire or urge to use drugs that you found hard to resist?	☐	☐	☐	☐	☐
2.3.4 How often in the last 6 months has it happened that you have not been able to stop taking drugs once you started?	☐	☐	☐	☐	☐
2.3.5 How often in the last 6 months did you fail to do what was normally expected of you because of drug use?	☐	☐	☐	☐	☐
2.3.6 How often in the last 6 months have you had to take drugs first thing in the morning to get yourself going after a heavy drug use session the day before?	☐	☐	☐	☐	☐
2.3.7 How often in the past 6 months have you had a feeling of guilt or remorse after using drugs?	☐	☐	☐	☐	☐
				**SCORE**	=

↓*For respondents who report substance use in PAST 6 MONTHS*

	**Yes, during the last 6 months**	**Yes, but not in the last 6 months**	**No**
2.3.8 Have you become involved in fights while under the influence of drugs?	☐	☐	☐
2.3.9 Have you engaged in sex for money, goods or drugs in order to obtain drugs?	☐	☐	☐
2.3.10 Have you engaged in illegal activities in order to obtain drugs?	☐	☐	☐
2.3.11 Have you been arrested for possession of illegal drugs?	☐	☐	☐
2.3.12 Have you ever had blackouts or flashbacks as a result of using drugs?	☐	☐	☐
2.3.13 Have you ever experienced withdrawal symptoms (felt sick) when you stopped taking drugs?	☐	☐	☐
2.3.14 Have you ever had medical problems as a result of your drug use (e.g. memory loss, fits, bleeding or hepatitis)?	☐	☐	☐
2.3.15 Have you or someone else been mentally or physically hurt because you used drugs?	☐	☐	☐
2.3.16 Has a relative, friend, doctor or other health care worker been concerned about your drug use or suggested that you should stop?	☐	☐	☐
2.3.17.a Have you ever injected drugs?	☐	☐	☐
→2.3.17.b IF **YES**, how often did you inject drugs in the past 6 months?		Once weekly or less ☐<br>More than once per week ☐	

2.4 Violence

You are doing really well and we are about three-quarters of the way through the interview. The information you have given us is very important.

There are now a few more questions about your experiences growing up, and your relationships with others. Some men find these questions hard to talk about, others find it easy. Remember that everything you share here will only be used for research purposes and will be kept strictly secret. We have not written down your name anywhere and what you say in this interview cannot be linked to you in any way, but it WILL help us a lot in understanding the lives of men in South Africa.

	**Never**	**Once**	**More than once**
2.4.1 While you were growing up, how often did someone in your household push, grab, shove, throw something, slap, hit, kick or punch you?	☐	☐	☐
2.4.2 While you were growing up, how often did your parents or the people who raised you push, grab, shove, throw something, slap, hit, kick or punch each other?	☐	☐	☐

→Go to 3. SEXUAL BEHAVIOUR AND PRACTICES

**3. SEXUAL BEHAVIOUR AND PRACTICES**		
I'd now like to ask you about your sexual partners and practices. While some of this information may be embarrassing or difficult to remember, please try and answer as truthfully as you can.		
3.1.1.a How many sexual partners have you had in the past 6 months?		☐☐ partners
→3.1.1.bIF >**1 partner**, did you have a relationship with more than one person at the same time during the last 6 months?	Yes ☐	No ☐
3.1.2. Some men have sex with other men. Have you had sex with another man in the last 6 months?	Yes ☐	No ☐
3.1.3 Do you identify yourself as?	Heterosexual/Straight ☐<br>Bisexual ☐<br>Other ☐	Homosexual/Gay ☐<br>→ Specify ______________
**3.2 Partners**		
I'd like to know more about ANY NEW sexual partner SINCE YOUR LAST VISIT, up to a maximum of 3 partners starting with your most recent sexual partner.<br>*For these questions, sex is defined as any vaginal or anal sex that is where a man inserts his penis into the vagina or anus. A sexual partner is someone with whom you have had sex either regularly or only once.*		
**3.2.1 Most recent *NEW* partner**		
3.2.1.a How old is this person?	Years ☐☐	
3.2.1.b Is this person male or female?	Female ☐	Male ☐
3.2.1.c How would you characterise your relationship with this person?<br>***Main partner*** =*person you regularly have sex with AND married to/living with*<br>***Regular partner*** = *person you regularly have sex with but not married/not living together*<br>***Occasional partner*** = *person you had sex with once or twice*	Main partner ☐<br>Occasional partner ☐	Regular partner ☐
3.2.1.d Is this person a new partner, i.e. someone that you had sex with for the first time in the past 3 months?	Yes ☐	No ☐

3.2.1.e Is this partner a recent immigrant to South Africa (i.e. arrived within the last 3 years)?	Yes ☐<br>Don't know ☐	No ☐
3.2.1.f Has this person ever tested positive for HIV?	Yes ☐<br>Don't know ☐	No ☐
3.2.1.g How long did you know this person before you first had sex with them?	Minutes ☐☐<br>Hours ☐☐<br>Weeks ☐☐	Months ☐☐<br>Years ☐☐
3.2.1.h During the last 3 months, how many times did you have sex with this partner?	No. of times ☐☐☐	
3.2.1.i How often did you use a condom when you had sex?	Always ☐<br>>/= to half the time ☐	< half the time☐<br>Never ☐
3.2.1.j Did you drink alcohol the last time that you had sex with this partner (either during sex or up to two hours before you had sex)?	Yes ☐	No ☐
3.2.1.k Did this partner give you or receive from you money, drugs, food or a place to stay in exchange for sex the last time that you had sex?	Yes ☐	No ☐
3.2.1.l What type of sex did you have with this person?<br><br>→ ***MULTIPLE RESPONSES*** *possible*	**Type of Sex Yes No**<br>Oral received ☐☐<br>Oral performed ☐☐<br>Vaginal ☐☐<br>Receptive insertion of digits in anus ☐☐<br>Receptive insertion of objects in anus ☐☐	**Type of Sex Yes No**<br>Anal receptive ☐☐<br>Anal insertive ☐☐<br>Rimming received ☐<br>Rimming performed ☐
3.2.1.m Do you know or suspect that this person has other sexual partners?	Yes ☐	No ☐
3.2.1.n Did you have sex with any other partners in the past 3 months?	Yes ☐	No ☐<br>↓*IF **NO**, go to **8. Genital Hygiene Practices***
**3.2.2 Second most recent partner in past 6 months**		
3.2.2.a How old is this person?	Years ☐☐	

3.2.2.b Is this person male or female?	Female ☐	Male ☐
3.2.2.c How would you characterise your relationship with this person?<br>***Main partner*** *=person you regularly have sex with AND married to/living with*<br>***Regular partner*** *= person you regularly have sex with but not married/not living together*<br>***Occasional partner*** *= person you had sex with once or twice*	Main partner ☐<br>Occasional partner ☐	Regular partner ☐
3.2.2.d Is this person a new partner, i.e. someone that you had sex with for the first time in the past 3 months?	Yes ☐	No ☐
3.2.2.e Is this partner a recent immigrant to South Africa (i.e. arrived within the last 3 years)?	Yes ☐<br>Don't know ☐	No ☐
3.2.2.f Has this person ever tested positive for HIV?	Yes ☐<br>Don't know ☐	No ☐
3.2.2.g How long did you know this person before you first had sex with them?	Minutes ☐☐<br>Hours ☐☐<br>Weeks ☐☐	Months ☐☐<br>Years ☐☐
3.2.2.h During the last 3 months, how many times did you have sex with this partner?	No. of times ☐☐	
3.2.2.i How often did you use a condom when you had sex?	Always ☐<br>>/= to half the time ☐	< half the time ☐<br>Never ☐
3.2.2.j Did you drink alcohol the last time that you had sex with this partner – either during sex or up to two hours before you had sex?	Yes ☐	No ☐
3.2.2.k Did this partner give you or receive from you money, drugs, food or a place to stay in exchange for sex the last time that you had sex?	Yes ☐	No ☐
3.2.2.l What type of sex did you have with this person?<br>→***MULTIPLE RESPONSES*** *possible*	**Type of Sex Yes No**<br>Oral received ☐☐<br>Oral performed ☐☐<br>Vaginal ☐☐	**Type of sex Yes No**<br>Anal receptive ☐☐<br>Anal insertive ☐☐

3.2.2.m Do you know or suspect that this person has other sexual partners?	Yes ☐	No ☐
3.2.2.n Did you have sex with any other partners in the past 3 months?	Yes ☐	No ☐<br>→*IF **NO**, go to **4.Genital Hygiene Practices***
**3.2.3 Third most recent partner in past 6 months**		
3.2.3.a How old is this person?	Years ☐☐	
3.2.3.b Is this person male or female?	Female ☐	Male ☐
3.2.3.c How would you characterise your relationship with this person?<br>***Main partner*** *=person you regularly have sex with AND married to/living with*<br>***Regular partner*** *= person you regularly have sex with but not married/not living together*<br>***Occasional partner*** *= person you had sex with once or twice*	Main partner ☐<br>Occasional partner ☐	Regular partner ☐
3.2.3.d Is this person a new partner, i.e. someone that you had sex with for the first time in the past 6 months?	Yes ☐	No ☐
3.2.3.e Is this partner a recent immigrant to South Africa (i.e. arrived within the last 6 years)?	Yes ☐<br>Don't know ☐	No ☐
3.2.3.f Has this person ever tested positive for HIV?	Yes ☐<br>Don't know ☐	No ☐
3.2.3.g How long did you know this person before you first had sex with them?	Minutes ☐☐<br>Hours ☐☐<br>Weeks ☐☐	Months ☐☐<br>Years ☐☐
3.2.3.h During the last 36months, how many times did you have sex with this partner?	No. of times ☐☐	
3.2.3.i How often did you use a condom when you had sex?	Always ☐<br>>/= to half the time ☐	< half the time☐<br>Never ☐

3.2.3.j Did you drink alcohol the last time that you had sex with this partner – either during sex or up to two hours before you had sex?	Yes ☐	No ☐
3.2.3.k Did this partner give you or receive from you money, drugs, food or a place to stay in exchange for sex the last time that you had sex?	Yes ☐	No ☐
3.2.3.l What type of sex did you have with this person?<br>→***MULTIPLERESPONSES*** *possible*	Type of sex Yes No<br>Oral received ☐☐<br>Oral performed ☐☐<br>Vaginal ☐☐	Type of Sex Yes No<br>Anal receptive ☐☐<br>Anal insertive ☐☐
3.2.3.m Do you know or suspect that this person has other sexual partners?	Yes ☐	No ☐

→Go to 4. GENITAL HYGIENE PRACTICES

4. GENITAL HYGIENE PRACTICES

Now I'm going to ask you some questions about your personal hygiene practices. While some of these questions may be embarrassing or difficult to answer, please try and answer as truthfully as you can.

4.1.a Are you circumcised?
Yes ☐ → Age of circumcision ☐☐
No ☐

→4.1.b Where were you circumcised?
Traditional initiation school ☐ Other religious setting ☐
Health care facility ☐ Don't know ☐

4.2.a Do you clean your penis before and/or after you have sex?
Yes, before sex ☐
Yes, before and after sex ☐
Yes, after sex ☐
No ☐ →*IF **NO**, go to 4.4.a*

→4.2.b IF **YES**, what do you use to clean your penis before and/or after sex?

→*Tick **MULTIPLE RESPONSES** if applicable*

**Substances**	**Yes**	**No**	**Substances**	**Yes**	**No**
Water	☐	☐	Other household products	☐	☐
Water and soap	☐	☐	Cloth/towel to wipe	☐	☐
[mbiza/herbs/powders	☐	☐			

4.3 How often do you clean your penis at the time of sex?
Always ☐
More than half of sex acts ☐
Less than half of sex acts ☐

4.4.a Did you use any substances to enhance your sexual performance?
Yes ☐ No ☐

→7.4.bIF **YES,** what products did you use to enhance your sexual performance?
Substances taken orally ☐ Substances applied to penis ☐
Pills ☐
Other ☐ →Specify________________

(Substances: Imbiza/muti/herbs/powders)

4.5 Do you know whether your sexual partner/s use products other than water to clean or tighten his/her genital area before and/or after sex?
Yes ☐ No ☐
Don't know ☐

→Go to 5.

5. SEXUAL HEALTH HISTORY

5.1 Have you ever had a sexually transmitted infection? Yes ☐ No ☐ Don't know ☐

5.2 In the last 6 months, has a health care provider other than someone from the study, diagnosed you with or treated you for any of the following sexually transmitted or genital infections:

		**Yes**	**No**	**Don't know**
	Burning urine or discharge from the penis	☐	☐	☐
	Genital ulcer disease (including genital herpes)	☐	☐	☐
	Swollen glands in the groin (Bubo)	☐	☐	☐
	Pain or swelling of testicles	☐	☐	☐
	Genital warts	☐	☐	☐
NA ☐	For **MSM**: Pain or discharge from the rectum	☐	☐	☐
	Syphilis (by serological test)	☐	☐	☐

5.3 Were you diagnosed with a sexually transmitted infection but don't know the name or it is not listed above? Yes ☐ → Details ______ No ☐ Don't know ☐

5.4 Were you given treatment because your partner had a sexually transmitted infection? Yes ☐ No ☐ Don't know ☐

5.5 Have you sought care from anywhere other than a government clinic in the past 6 months? Yes ☐ → Details ______ No ☐ → Go to Question 5.6

5.6 IF you received treatment, did you:

→5.6.a Complete your treatment as prescribed? Yes ☐ No ☐

→5.6.b Have sex while taking treatment? Yes ☐ No ☐

→5.6.cIF **you had sex while taking treatment,** did you use a condom for every sex act?

Yes ☐ No ☐

→5.6.d Were all your partners treated? Yes ☐ No ☐ Don't know ☐ Not applicable ☐

→5.6.e Do you still have any genital symptoms? Yes ☐ No ☐ Not applicable ☐

5.7 In the past 7 days, have you experienced:	Yes	No	Don't Know
Burning urine or discharge from the penis	☐	☐	☐
Blisters or sores in the genital area	☐	☐	☐
Swollen glands in the groin (Bubo)	☐	☐	☐
Pain or swelling of testicles	☐	☐	☐
Genital warts	☐	☐	☐
NA ☐ For **MSM**: Pain or discharge from the rectum	☐	☐	☐
5.8 In the past 7 days have you been given treatment because your partner has an STI	☐	☐	☐

→Go to 6. PHYSICAL EXAMINATION

6. PHYSICAL EXAMINATION

6.1 Body Temperature	☐☐.☐ °C	
6.2 Weight	☐☐☐ kilograms	
6.3 Heart Rate	☐☐☐ per minute	
6.4 Blood Pressure	Systolic: ☐☐☐ mmHg	Diastolic: ☐☐☐ mmHg

**6.5 Body system or Part Observations**	**Not Examined**	**Normal**	**Abnormal**	***→IF ABNORMAL, details below:***
a. Skin and mucous membranes	☐	☐	☐	
b. Head and neck	☐	☐	☐	
c. Cardiovascular	☐	☐	☐	
d. Respiratory	☐	☐	☐	
e. Gastrointestinal (abdominal & liver)	☐	☐	☐	
f. Neurological	☐	☐	☐	
g. Genital	☐	☐	☐	*(see below)*

**6.6. Genital Examination Observations**	**Not Examined**	**No**	**Yes**	***→IF YES details below:***
a. Is participant circumcised?	☐	☐	☐	Partial ☐ Full☐
b. Inguinal lymphadenopathy?	☐	☐	☐	Single ☐ Multiple ☐ Bubo ☐
c. Balanitis?	☐	☐	☐	
d. Discharge?	☐	☐	☐	**Site Yes No** Meatal ☐☐ **Site Yes No** Subpreputial☐☐
e. Ulcers?	☐	☐	☐	
IF **YES**,	→ Ulcer Number:			Single ☐ Multiple ☐
	→ Ulcer Location (*Tick multiple locations if applicable*):			Prepuce ☐ Glans ☐ Shaft ☐ Inguinal ☐ Buttocks ☐ Perianal ☐ Other ☐

	If there are Ulcers and participant has consented,	→ Ulcer Pain: Yes ☐ No ☐<br>→ Ulcer Bleeding: Yes ☐ No ☐			
f.	Warts?	☐	☐	☐	Single ☐ Multiple ☐<br>Location:______________________<br>**If there are warts and consent take a photo**
g.	Peri-anal abnormalities?	☐	☐	☐	Details,_______________________<br>**If yes and consent take a photo**
h.	Other genital abnormality or deformity?	☐	☐	☐	Details,_______________________<br>___<br>**If yes and consent take a photo**

→Go to 7. LABORATORY SAMPLE COLLECTION

**7. Samples collected**	
**Blood samples**	
**HIV PVL**<br>**CD4+ cell count**	**Yes**☐ **No** ☐<br>**Yes**☐ **No** ☐
**HBsAntigen**	**Yes**☐ **No** ☐
**Syphilis serology:**<br>**RPR**<br>**TPHA**	 **Yes**☐ **No** ☐<br>**Yes**☐ **No** ☐
**HSV – 2 serology**	**Yes**☐ **No** ☐
**HPV serology**	**Yes**☐ **No** ☐
**Urine samples**	
***C. trachomatis* PCR**	**Yes**☐ **No** ☐
***N. gonorrhoeae* PCR**	**Yes**☐ **No** ☐
***T. vaginalis* PCR**	**Yes**☐ **No** ☐
***M. genitalium* PCR**	**Yes**☐ **No** ☐
**Genital samples**	
**HPV genital swab**	**Yes**☐ **No** ☐
**HPV anal canal swab**	**Yes**☐ **No** ☐
**Anal cytology swab**	**Yes**☐ **No** ☐
**Oral samples**	
**oral rinse sample**	**Yes**☐ **No** ☐
**oral HPV swab**	**Yes**☐ **No** ☐

Go to 8 Diagnosis and treatment

8. DIAGNOSIS AND TREATMENT		
IF REQUIRED, please provide participant with ***script for treatment*** *to be collected at the pharmacy and* ***partner notification slips.***		
8.1 Was the participant treated presumptively for any of the following at this visit?	**Yes**	**No**
Urethritis syndrome	☐	☐
Genital ulcer syndrome	☐	☐
Scrotal swelling	☐	☐
Balanitis / Balanoposthitis	☐	☐
Bubo	☐	☐
Molluscumcontagiosum	☐	☐
Genital Warts	☐	☐
Pubic lice	☐	☐
	**If Yes to any complete the concomitant Log**	
8.2.a Did the participant require treatment for ANY OTHER CONDITION?	Yes ☐<br>**If yes to any complete the concomitant Log**	No ☐
→8.2.b IF **YES**, provide details		
