# Supplementary material for: Prevalent, persistent anal HPV infection and squamous intraepithelial lesions: Findings from a cohort of men living with HIV in South Africa
Source: PLoS One. 2019 Dec 5;14(12):e0225571. doi: 10.1371/journal.pone.0225571 (PMC6894774; doi:10.1371/journal.pone.0225571)
Supplement: S4 File — (PDF) [file pone.0225571.s006.pdf]

strate, per(100)

```
        failure _d:  hpvan_anyinc == 1
  analysis time _t:  (v3date-origin)/365.25
            origin:  time vdateic
 enter on or after:  time vdateic
                id:  ptid
```

Estimated rates (per 100) and lower/upper bounds of 95% confidence intervals

(180 records included in the analysis)

```
  +-----------------------------------------+
  | D       Y     Rate     Lower     Upper |
  |-----------------------------------------|
  | 2   2.5364   0.78853   0.19721   3.15288 |
  +-----------------------------------------+
```
